# Fractality and Variability in Canonical and Non-Canonical English Fiction and in Non-Fictional Texts

**DOI:** 10.3389/fpsyg.2021.599063

**Published:** 2021-03-31

**Authors:** Mahdi Mohseni, Volker Gast, Christoph Redies

**Affiliations:** ^1^Experimental Aesthetics Group, Institute of Anatomy I, Jena University Hospital, University of Jena, Jena, Germany; ^2^Department of English and American Studies, University of Jena, Jena, Germany

**Keywords:** fractality, self-similarity, multifractal DFA, variability, POS tagging, sentence length, lexical diversity, topic modeling

## Abstract

This study investigates global properties of three categories of English text: canonical fiction, non-canonical fiction, and non-fictional texts. The central hypothesis of the study is that there are systematic differences with respect to structural design features between canonical and non-canonical fiction, and between fictional and non-fictional texts. To investigate these differences, we compiled a corpus containing texts of the three categories of interest, the Jena Corpus of Expository and Fictional Prose (JEFP Corpus). Two aspects of global structure are investigated, variability and self-similar (fractal) patterns, which reflect long-range correlations along texts. We use four types of basic observations, (i) the frequency of POS-tags per sentence, (ii) sentence length, (iii) lexical diversity, and (iv) the distribution of topic probabilities in segments of texts. These basic observations are grouped into two more general categories, (a) the lower-level properties (i) and (ii), which are observed at the level of the sentence (reflecting linguistic decoding), and (b) the higher-level properties (iii) and (iv), which are observed at the textual level (reflecting comprehension/integration). The observations for each property are transformed into series, which are analyzed in terms of variance and subjected to Multi-Fractal Detrended Fluctuation Analysis (MFDFA), giving rise to three statistics: (i) the degree of fractality (H), (ii) the degree of multifractality (D), i.e., the width of the fractal spectrum, and (iii) the degree of asymmetry (A) of the fractal spectrum. The statistics thus obtained are compared individually across text categories and jointly fed into a classification model (Support Vector Machine). Our results show that there are in fact differences between the three text categories of interest. In general, lower-level text properties are better discriminators than higher-level text properties. Canonical fictional texts differ from non-canonical ones primarily in terms of variability in lower-level text properties. Fractality seems to be a universal feature of text, slightly more pronounced in non-fictional than in fictional texts. On the basis of our results obtained on the basis of corpus data we point out some avenues for future research leading toward a more comprehensive analysis of textual aesthetics, e.g., using experimental methodologies.

## 1. Introduction

Canonical fiction comprises works “which are accepted as legitimate by the dominant circles within a culture and whose conspicuous products are preserved by the community to become part of its historical heritage” (Even-Zohar, 1990, p. 15); they are regarded as “repositories of cultural values” (Guillory, 1987, p. 487). The relevant texts have high prestige (“classics,” “high literature”) and are often integrated into the school curriculum, so that large parts of a society are familiar with them. In this study we investigate whether English canonical and non-canonical texts from the 19th and early 20th centuries differ in terms of global structural design features. In order to locate the two text categories in the larger space of genres, we moreover compare fictional texts with non-fictional texts. The study is embedded within the field of empirical textual aesthetics insofar as the three text categories under analysis differ in terms of either the presence or absence of an aesthetic function (fictional vs. non-fictional texts), or preferences of societies as reflected in canonization. While canonization is a process driven by a range of social variables, such as “publication mechanisms (i.e., the sale of books, library use, etc.), politics, etc.,” it is also based on “the text, its reading, readership, literary history, [and] criticism,” i.e., the work of art itself in its cultural context (Tötösy de Zepetnek, 1994, p. 109, cf. also Underwood and Sellers, 2016; Koolen et al., 2020 for a discussion of the relationship between text-intrinsic and text-extrinsic factors in the process of canonization). The question arises whether there are any measurable differences between the text categories of interest. In this article we address this question by analyzing texts in terms of fractality and variability.

The question of objective, measurable correlates of readers' or societies' attitudes to texts has been raised in various contexts, more or less explicitly. The assumption that artistic composition can be measured is most obvious for poetry, with its interplay of meaning and form as manifested in rhythm and rhyme, and other aspects of poetic form, e.g., alliteration (cf. for instance Jakobson, 1960; Leech, 1969; Jacobs, 2015; Jacobs et al., 2016; Vaughan-Evans et al., 2016; König and Pfister, 2017; Menninghaus et al., 2017; Egan et al., 2020; Menninghaus and Wallot, 2021). Relevant studies of prose have mostly used summary statistics of properties extracted from text. Louwerse et al. (2008), one of the earliest relevant studies from the field of computational linguistics, distinguished literary from non-literary texts with distance measures derived from Latent Semantic Analyses and fed into a hierarchical clustering algorithm, and with frequency distributions of unigrams and bigrams. van Cranenburgh and Bod (2017) used frequency distributions of lexical and syntactic features to model human ratings of texts as more or less “literary” (see also Ashok et al., 2013; van Cranenburgh and Koolen, 2015 for similar approaches). In van Cranenburgh et al. (2019), summary statistics derived from topic modeling (Latent Dirichlet Allocation) and paragraph vectors are used to predict degrees of “literariness.” Maharjan et al. (2017) explore a wide variety of features (including “readability”) that can be used to classify texts in terms of “likability.” Other standard methods of computational linguistics used in this context include sentiment and emotion analysis (Alm and Sproat, 2005; Francisco and Gervás, 2006; Kakkonen and Galić Kakkonen, 2011; Mohammad, 2011; Reagan et al., 2016; Maharjan et al., 2018). Global statistical properties such as complexity and entropy have been used to study the regularity (Mehri and Lashkari, 2016; Hernández-Gómez et al., 2017) and the quality of texts (Febres and Jaffe, 2017). Fractal analysis, which figures centrally in our study, has been applied to fictional texts as well (Drożdż and Oświȩcimka, 2015; Mehri and Lashkari, 2016; Chatzigeorgiou et al., 2017), and fractal patterns have been observed in both Western (Drożdż et al., 2016) and Chinese literature (Yang et al., 2016; Chen and Liu, 2018). Cordeiro et al. (2015, p. 796) claim that “there is a fractal beauty in the text produced by humans” and “that its quality is directly proportional to the degree of self-similarity.”

Our approach to studying structure in texts is inspired by relevant findings from vision, which we take as our starting point. In (cognitive) linguistics there is a widespread assumption that “linguistic structure is shaped by domain-general processes” (Diessel, 2019, p. 23), such as figure-ground segregation and processes of memory retrieval. In other words, linguistic processing is assumed to be based on the same type of brain activity as the processing of other types of sensory input. We therefore use methods that have been successfully applied in vision for the analysis of textual data. This transfer has obvious limitations though. Image data are three-dimensional—two-dimensional matrices with the luminance/color signals as the third dimension—whereas textual data are *prima facie* one-dimensional when regarded as strings of characters (though even silent reading implies prosody, adding a second dimension, cf. Gross et al., 2014). Related to this, the processing of propositional information is an incremental, “piecewise buildup of information, adding bits of information as the reader advances through the text” (Wallot et al., 2014, p. 1748; see also Verhuizen et al., 2019). Reading a text is thus a less immediate experience than contemplating a picture, in the sense that it requires more higher-level activity. Still, the higher-level activity of integrating new information into a “situation model” (Kintsch, 1988; McNamara and Magliano, 2009; Zwaan, 2016) is fed by lower-level processes of linguistic decoding (Cain et al., 2004; Tiffin-Richards and Schroeder, 2015, 2018)^1^.

We use vision as our point of reference because there is a long-standing tradition of empirical research on aesthetic perception in this domain (Fechner, 1876; Arnheim, 1974; Chatterjee and Vartanian, 2014; Jacobs, 2015; Redies, 2015), and artworks have been studied in terms of structural properties (for reviews, see Taylor et al., 2011; Brachmann and Redies, 2017). In this work, objective image properties were identified that differ between various categories of man-made images, such as traditional visual artworks, other visually preferred images and different types of non-preferred images. A particular focus has been on global properties of preferred stimuli. In contrast to local image properties, such as luminance contrast or color at a given location in an image, global image properties reflect summary statistics of pictorial elements or their relations to each other across an image (Brachmann and Redies, 2017). Global statistical image properties seem particularly suitable for studying visual preferences because aesthetic concepts, such as “balanced composition” (McManus et al., 1985), “good Gestalt” (Arnheim, 1974), or “visual rightness” (Locher et al., 1999) all refer to global image structure (Redies et al., 2017). Examples of global properties that characterize preferred visual stimuli are a scale-invariant (fractal) image structure (Taylor et al., 2011), statistical regularities in the Fourier domain (Graham and Field, 2007; Redies et al., 2007), curved shape (Bar and Neta, 2006; Bertamini et al., 2016), regularities in edge orientation distribution (Redies et al., 2012, 2017), and specific color features (Palmer et al., 2013; Nascimento et al., 2017). Moreover, traditional visual artworks were found to exhibit a high richness and high variability of low-level features of a Convolutional Neural Network (CNN; Brachmann et al., 2017).

Given the time-distributed nature of information processing in reading, aesthetic experience is hard to measure experimentally in this domain (see e.g., Cook and Wei, 2019 for discussion). Studies obtaining real-time measurements (such as reading times) generally investigate smaller windows of text (e.g., O'Brien et al., 2013; Wallot et al., 2014; Blohm et al., 2021; Menninghaus and Wallot, 2021)^2^. The methods used in vision research can thus not easily be transferred to the study of aesthetic experience in reading. In this study we therefore pursue an observational, rather than experimental approach, investigating properties of texts which are classified along the dimensions fictional/non-fictional and (within the fictional texts) canonical/non-canonical.

As a first step, we need to identify measurable properties that differentiate fictional from non-fictional texts, and canonical from non-canonical fictional texts. Moreover, we need to test and validate statistical methods to describe global structural patterns in the distribution of these properties in texts. We will use two text properties that we regard as being relevant to (linguistic) decoding, measurements derived from part-of-speech tags and sentence length, and two properties that we regard as correlates of higher-level comprehension processes, lexical diversity and topic probabilities. For each of these properties, which are represented as series, we determine four statistics reflecting variability and fractality, the most important determinants distinguishing visual artworks of different categories (Redies and Brachmann, 2017)^3^. For our quantitative analysis we have compiled a corpus of fictional and non-fictional texts, the Jena Corpus of Expository and Fictional Prose, JEFP Corpus for short. The fictional texts of this corpus are classified into canonical and non-canonical ones (see section 4 for details). Obviously, our observational approach does not allow us to reach any conclusions concerning cognitive processes during reading (aesthetic experience, e.g., aesthetic emotions as described by Menninghaus et al., 2019), and we abstract away from the role of the reader (see Iser, 1976 for a foundational study of aesthetic responses during reading, and recent empirical studies of the type carried out by Blohm et al., 2021; Menninghaus and Wallot, 2021). We therefore also disregard phonological aspects of texts, which are no doubt important for aesthetic textual perception. Our study is intended to provide the basis for experimental investigations in the future by identifying textual properties, and global patterns in the distribution of such properties, that vary across the text types distinguished in this study.

The article is organized as follows: We start by providing a list of measurable text properties that may contribute to differences between the three categories of texts in the corpus (section 2). Based on these properties, series are derived from the various texts. We then proceed to introduce statistical methods that capture variability and fractal patterns, most importantly Multi-Fractal Detrending Fluctuation Analysis (MFDFA, section 3). The Jena Corpus of Expository and Fictional Prose (JEFP Corpus) is described in section 4. In section 5, we provide the results of individual features relative to the three text categories and we show how well they can distinguish between the categories by feeding them into a binary classifier (Support Vector Machine). In section 6, we discuss the implications of our preliminary findings and outline avenues for future research.

## 2. Measurable Properties of Text

The central hypothesis of this study is that texts of the categories fictional/canonical, fictional/non-canonical, and non-fictional differ in terms of measurable structural properties. Such properties can be derived from various types of measurements. While we are ultimately interested in global properties of texts, the basic units of observations are located at different levels of processing. As mentioned in section 1, we distinguish two levels of processing. The lower level of processing concerns the task of linguistic decoding, which is largely automatic and resorts to implicit knowledge. The higher level of processing concerns the integration of propositional information into explicit memory (comprehension).

While the lower-level processes of reading have been studied experimentally in psychological, psycholinguistic and neurolinguistic research, e.g., with eye-tracking and event-related potential measurements (e.g., Kliegl et al., 2004, 2012), comprehension has been studied most extensively in the field of the psychology of learning, specifically in text assessment (e.g., Graesser et al., 2003; McNamara et al., 2013). The Coh-Metrix tool, which “analyzes texts on over 200 measures of cohesion, language, and readability” (Graesser and Kulikowich, 2011, p. 193) has been developed for the analyses of texts at the higher level of processing, e.g., by focusing on coherence and cohesion. Given the wide range of text properties that have been used as correlates of behavioral measurements in various fields (e.g., computational linguistics and the psychology of learning), in this exploratory study we can only focus on a selection of properties that we expect to be relevant to our research programme. We use two types of lower-level properties (frequencies of part-of-speech tags and sentence length) and two types of higher-level properties (lexical diversity and topic distribution). This is not of course to say that other properties are not potentially relevant to our research programme. Building upon our results we intend to explore additional properties in the future, both from studies on readability (e.g., the measurements delivered by Coh-Metrix) and from Natural Language Processing, e.g., language modeling^4^ and embedding vectors^5^.

In what follows we briefly characterize the four text properties used for our study, without providing any technical details. The derivation of series on the basis of these properties is described in section 5.1.

**Part-of-speech tags**, commonly abbreviated as “POS-tags,” represent the syntactic class of a word. To some extent, they reflect syntactic structure. At the most general level, POS-tags classify words into major classes, such as “noun,” “verb,” “adjective,” etc., but depending on the specific tagset used, more fine-grained distinctions can be made (e.g., between singular and plural nouns). Parts of speech are considered to be potentially relevant to our research programme because they provide important categorical information at the word level, which is no doubt prominent in reading because text is primarily structured into words, separated by white spaces. Accordingly, “lexical variables are thought to be the main driving force behind the reading process” (Wallot et al., 2014, p. 1746) (note that Wallot et al., 2014, p. 1746 actually reach the conclusion that “lexical features do not play a substantial role in connected text reading,” but they only took word length and frequency into account, no categorical information; see also Wallot et al., 2013). Moreover, neurological studies have shown that different parts of speech, e.g., nouns, verbs, and adjectives, are processed at different cortical locations (Perani et al., 1999; Tyler et al., 2004; Scott, 2006; Shapiro et al., 2006; Cappelletti et al., 2008; Sudre et al., 2012; Fyshe et al., 2019). We have no precise expectation with respect to the type of effect that part-of-speech distributions may have on reading processing, or how their distributions may vary across the text categories compared in this study. We do expect them to be potential correlates of reading experience, however, e.g., because they differ in terms of their informativeness (see Seifart et al., 2018 for evidence showing that nouns are more informative than verbs, requiring more cognitive resources), and the type of information that they convey. For our study, we used the Stanford Tagger (version 3.6.0; see section 5.1 for details).

**Sentence length**, measured in terms of the number of tokens in a sentence, is a very basic indicator of lower-level text structure. In fictional texts, it is potentially informative because it tends to differ between narrative passages (with longer sentences) and passages with dialogues (with shorter sentences). The distribution of sentence length values across a text therefore, to some extent, reflects the text's composition in terms of perspective (external communication with narrative elements vs. internal communication, e.g., dialogs, monologs, thoughts). Sentence length was used in earlier approaches to text assessment (see for instance Petersen, 2007), and it has been used as a measurement for the study of fractality before by Drożdż et al. (2016), though not for a comparison of text types. Even though sentence length is certainly a rough indicator of lower-level text structure, it provides a starting point before we apply more specific measures^6^.

**Lexical diversity**, a derivative of the choice of words in a text, is one of the most perspicuous text properties, and a rich vocabulary is often regarded as a hallmark of good authorship. For example, Simonton (1990) claims that lexical diversity correlates with “aesthetic success.” He analyzed Shakespeare's sonnets and showed that there is a vocabulary shift from the more “obscure” to the more popular sonnets. Vocabulary and the richness of lexicon has also been found useful in the assessment of writers' proficiency, for instance in research on second language acquisition (see Laufer and Nation, 1995; Zareva et al., 2005; Yu, 2009). Given the importance of lexical diversity for readability measures, it is natural to include it in a study analyzing text properties that can be expected to have correlates in aesthetic experience. As a measurement of lexical diversity, we have used MTLD (see McCarthy and Jarvis, 2010 and section 5.1).

**Topic modeling** is a method used to analyze the content of texts by revealing hidden topics of documents in a collection. It has been used in computational studies of literary texts before, though with different objectives and background assumptions (van Cranenburgh et al., 2019). We are interested in the changes of topic distributions along a text, as it can be expected to have an impact on how “a reader progresses through a text with a growing understanding for its content, topics and themes” (Wallot et al., 2014, p. 1749). To extract the distribution of topics from a text, the text is split into segments and then, to infer the topic distribution, a topic modeling method is applied (using Latent Dirichlet Allocation/LDA, see section 5.1).

## 3. Global Measures of Variability and Self-Similarity

In the present section, we introduce ways of analyzing the series of text properties that were introduced in the previous section. We focus on two global statistical features (variability and self-similarity). These properties were selected because they have previously been used in visual aesthetics and have been shown to be associated with artworks and other visually pleasing stimuli (see section 1).

Variability reflects the degree to which a particular feature (e.g., edge orientation or color) is likely to vary across an image. It can be measured simply by computing the variance of a series. The variance of a random variable *X* is

(1) $$\begin{array}{r} V\left(X\right)=E\left[{\left(X-\mu \right)}^{2}\right] \end{array}$$

*E*[.] denotes the expected value and μ is the population mean. The variance of, for example, the distribution of sentence length reflects the amount of variation in the length of sentences across a text. Despite its mathematical simplicity, we will see that variance performs effectively in the classification of text categories (section 5).

Fractality and self-similarity reflect the degree to which parts of an image have features similar to the image as a whole, i.e., an image is self-similar if it shows similar features at different scales of resolution (scale-invariance). To analyze variability and fractality/self-similarity, several methods are available. The method used in the present study (Multi-Fractal Detrended Fluctuation Analysis/MFDFA) is described below. Alternative methods, such as methods based on entropy, box counting, wavelets and cross-correlation analysis, are described in the Supplementary Material.

### 3.1. Multi-Fractal Detrended Fluctuation Analysis

Self-similarity can be measured with Detrended Fluctuation Analysis (DFA) (Peng et al., 1994) and its extension Multi-Fractal DFA (MFDFA) (Kantelhardt et al., 2002; Oświecimka et al., 2006). These methods have been widely used for studying long-range correlations in a broad range of research fields, such as biology (Das et al., 2016), economics (Caraiani, 2012), music (Sanyal et al., 2016), and animal song (Roeske et al., 2018). MFDFA can be related to Fourier spectral analysis and both methods provide similar results for the degree of fractality (Heneghan and McDarby, 2000). Moreover, MFDFA has a theoretical and practical connection to wavelet-based methods (Leonarduzzi et al., 2016).

In the present work, we will apply MFDFA to the fractal analysis of texts. MFDFA has been used for textual analysis before. For example, Drożdż and Oświȩcimka (2015) applied this method to sentence-length series in comparison to other natural series (e.g., the discharge of the Missouri river and sunspot number variability) and non-natural series (e.g., stock market and Forex index prices). The results suggest that natural languages possess a multifractal structure that is comparable to that of other natural and non-natural phenomena. Yang et al. (2016) investigated long-range correlations in sentence-length series in a famous classic Chinese novel, based on the number of characters in each sentence. This study showed that there was a long-range correlation, though it was weak. A diachronic fractality analysis of word-length in Chinese texts spanning 2,000 years revealed two different long-range correlations regimes for short and large scales (Chen and Liu, 2018). An analysis of fractality of sentence-length series in several Western fictional texts revealed that, although most fictional texts show a long-range correlation, the degree of multifractality can vary quite substantially, ranging from monofractal to highly multifractal structure (Drożdż et al., 2016). Although sentence length can be measured in various ways, e.g., as the number of characters or words in unlemmatized and lemmatized texts, the different ways yield robust results that have comparable distributions and similar patterns of long-range correlations (Vieira et al., 2018). MFDFA has also been applied in empirical studies of reading (Wallot et al., 2014).

Given a series *X* = *x*_1_, *x*_2_, ⋯ , *x*_*N*_, MFDFA can be summarized as follows:

1. Subtract the mean and compute the cumulative sum, called the profile, of the series: $Y\left(i\right)=\overset{i}{\underset{k=1}{\sum }}\left[x_{k}-\langle x\rangle \right],i=1,\cdots \;,N$
2. Divide the profile of the signal into *N*_*s*_ = *N*/*s* windows for different values of *s*
3. Compute the local trend, *Y*′, which is the best fitting line (or polynomial), in each window
4. Calculate the mean square fluctuation of the detrended profile in each window *v*, *v* = 1, ⋯ , *N*_*s*_ : $F^{2}\left(s,v\right)=\frac{1}{s}\overset{s}{\underset{i=1}{\sum }}{\left[Y\left(s\times \left(v-1\right)+i\right)-Y^{\prime }\left(s\times \left(v-1\right)+i\right)\right]}^{2}$
5. Calculate the *q*th order of the mean square fluctuation: $F_{q}\left(s\right)={\left\{\frac{1}{N_{s}}\overset{N_{s}}{\underset{v=1}{\sum }}{\left[F^{2}\left(s,v\right)\right]}^{q/2}\right\}}^{1/q}$
6. Determine the scaling behavior of *F*_*q*_(*s*) vs. *s*: $F_{q}\left(s\right)\sim s^{h\left(q\right)}$

In the windowing procedure, as the length of the series, *N*, is not usually divisible by the chosen window size, *s*, a part of the series may be ignored. Therefore, it is possible to repeat the windowing procedure, starting from the end. Accordingly, the number of segments rises up to 2 × *N*_*s*_, which is taken into account in the averaging in step 5. In our experiments, we analyzed each series in windows of size *s*_*i*_; *s*_0_ = 16 and $s_{i}=s_{i-1}+2^{\left\lfloor \log\left(s_{i-1}\right)-1\right\rfloor }$, for *i*≥1 and *s*_*i*_ ≤ ⌊*N*/3⌋. In other words, the size of the windows is selected from the sequence 16, 24, 32, 48, 64, …, up to a point where the series is split into three non-overlapping segments. Detrending is accomplished by linear fits, so the fluctuation is computed according to the deviation from the best fitted line in each window. We changed the parameter of the fluctuation function, *q*, from −5 to 5 with a step size of 0.25.

### 3.2. The Degree of Fractality

The procedure of MFDFA is equivalent to DFA if *q* is fixed at 2. For monofractal series, *h*(*q*) is independent of *q*. If a series is stationary, *h*(2) is equal to the Hurst Exponent, a well-known measure in fractal analysis studies. We refer to this value as H, the degree of fractality of the series. In the remainder of this text, wherever we use “Hurst exponent” we refer to this value, even though the series may not be stationary. For uncorrelated series, in which each event is independent of other events, H≃0.5. With H>0.5, the series is more fractal. In the opposite direction, if H<0.5, the series is called anti-persistent. In such cases a large value in the series is most likely followed by a small value, and vice versa.

**Figure 1:**
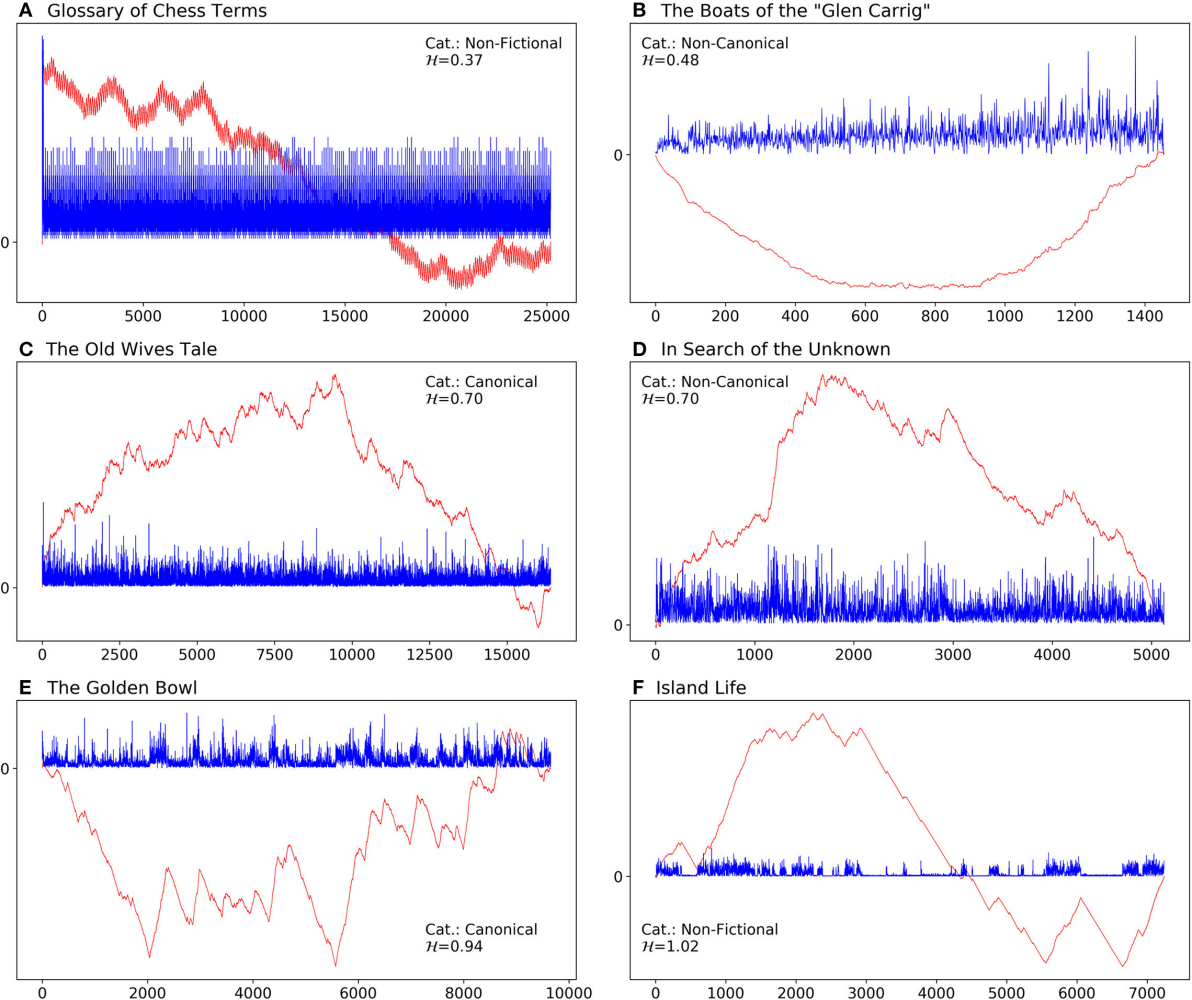
Sentence length series (blue) and their profiles (red; cumulative sum of mean centered series) of some example texts in the corpus. The series have been scaled up by a factor of 20 to show more detail. The category (Cat.) and the fractal degree, H, of each text is shown inside each panel. **(A)** *Glossary of Chess Terms* by Gregory Zorzos, with the lowest fractal degree in our corpus. **(B)** *Boats of the “Glen Carrig”* by William Hope Hodgson with H=0.48, a non-canonical text with the lowest value among the fictional books. **(C)** *Women in Love* by D. H. Lawrence, the median of canonical texts. **(D)** *In Search of the Unknown* by Robert W. Chambers, representing the median of non-canonical texts. **(E)** *The Golden Bowl* by Henry James, with the highest fractal degree among canonical texts. **(F)** *Island Life* by Alfred Russel Wallace, a non-fictional text, with the highest fractal degree in the whole corpus.

To get a more intuitive understanding of H, we show the sentence-length series of a few cases in our corpus (section 4) as well as the profile of each series in Figure 1 (see step 1 of MFDFA in above). Figure 1A represents the series of the *Glossary of Chess Terms* by Gregory Zorzos, which is one of the texts in the non-fictional categories of our corpus. This dictionary-like book consists of a list of terms and their definitions. It represents an example of an anti-persistent text, with H=0.37, and it is an extreme case in the corpus, with the lowest fractal degree. Figure 1B corresponds to *The Boats of the “Glen Carrig”* by William Hope Hodgson. With H=0.48, this book has the second lowest H value and is closest to 0.5, which shows that there is almost no correlation among the elements of its series. This book is categorized as a non-canonical text in our corpus. As a side note, the lower bound of fractality for sentence-length series of canonical texts in the corpus is at H=0.58, which is the value measured for *Old Mortality* by Walter Scott. In Figures 1C,D we show the plots of one canonical and one non-canonical fictional book with a medium degree of fractality, within the relevant category/sub-corpus. For both *The Old Wives' Tale* by Arnold Bennett, a canonical text, and *In Search of the Unknown* by Robert W. Chambers, a non-canonical text, H=0.70. Figure 1E represents the series of a canonical text with the highest fractal degree (H=0.94) in the corpus, namely *The Golden Bowl* by Henry James. Finally, the text with the highest value of H in the entire corpus is *Island Life* by Alfred Russel Wallace, a text from the non-fictional sub-corpus, with H=1.02.

### 3.3. The Degree of Multifractality and Fractal Asymmetry

From *h*(*q*), one can compute the degree of multifractality and the fractal asymmetry, two metrics that represent the fractal complexity of the series. From *h*(*q*), the Hölder exponents, α, and the singularity spectrum, *f*(α), are computed as follows (*h*′ is the derivative of *h*):

(2) $$\begin{array}{r} \alpha =h\left(q\right)+qh^{\prime }\left(q\right) \end{array}$$

(3) $$\begin{array}{r} f\left(\alpha \right)=q\left[\alpha -h\left(q\right)\right]+1 \end{array}$$

Then, the degree of multifractality is defined as $D=\alpha_{max}-\alpha_{min}$ (cf. Kantelhardt et al., 2002; Drożdż et al., 2016). α_*min*_ and α_*max*_ denote the beginning and the end of *f*(α), respectively. The fractal asymmetry is also computed from *f*(α):

(4) $$\begin{array}{r} A=\frac{\Delta \alpha_{L}-\Delta \alpha_{R}}{\Delta \alpha_{L}+\Delta \alpha_{R}} \end{array}$$

where Δα_*L*_ =α_0_−α_*min*_ and Δα_*R*_ = α_*max*_−α_0_ (Drożdż and Oświȩcimka, 2015). α_0_, corresponding to *q* = 0, usually points to the peak of the *f*(α) curve. It is obvious that $D=\Delta \alpha_{L}+\Delta \alpha_{R}$. In section 5, we will use the three values (fractal degree [H], degree of multifractality [D], and fractal asymmetry [A]) as a basis for classifying the three categories of text (canonical, non-canonical, and non-fictional).

**Figure 2:**
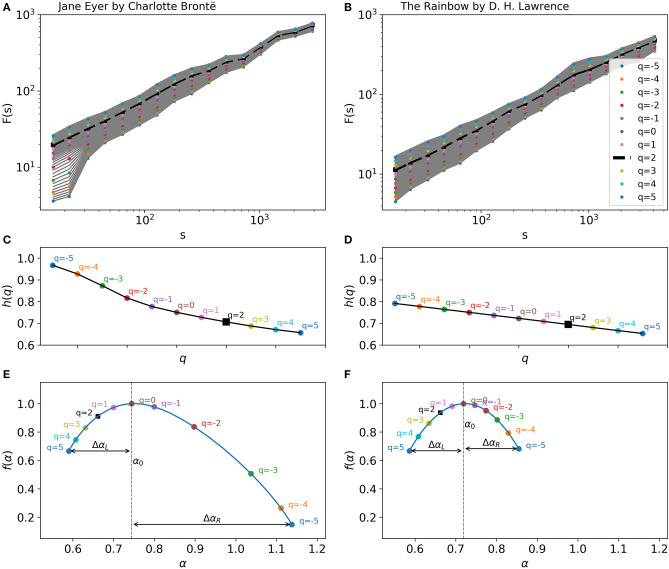
**(A,B)** *q*th order of mean square fluctuation of sentence-length series of *Jane Eyre* by Charlotte Brontë **(A)** and *The Rainbow* by D. H. Lawrence **(B)**. The plots show *F*_*q*_(*s*) for different scales of *s* and for different values of *q*, ranging from −5 to 5 in steps of 0.25. The colored points represent values that correspond to the integer *q*s. H is the slope of the best linear fit to the dashed curve, which corresponds to *q* = 2, and which identical to the output of the DFA method. **(C,D)** Slopes of the best linear fits to the fluctuation function in **(A,B)**, respectively. From these plots, singularity spectra are computed by the Legrand transformation. **(E,F)** Singularity spectra of the two texts. α_0_ indicates the peak of each curve. The width of the curve, Δα = Δα_*L*_+Δα_*R*_, known as the degree of multifractality (D), shows how multifractal a series is. Here, both texts are highly multifractal. The fractal asymmetry (A) of the curve is calculated from Δα_*L*_ and Δα_*R*_. The curve is asymmetrical for *Jane Eyre*, but symmetrical for *The Rainbow*.

To illustrate these concepts visually, we show the results of the fractal analysis for canonical texts by Charlotte Brontë and D. H. Lawrence in Figure 2. The two texts have been converted to series by using the sentence-length property. Figures 2A,B show *F*_*q*_(*s*) for different values of *q* ranging from −5 to 5. The slopes of the linear fits to the curves of *F*_*q*_(*s*) are represented in Figures 2C,D for the two texts, respectively. It is obvious that the slopes of the fits, *h*(*q*), change as *q* changes. This result indicates that the texts are multifractal.

By applying Equations (2) and (3) to these plots, the singularity spectrum of the series is computed as shown in Figures 2E,F. *Jane Eyre* by Charlotte Brontë has a high degree of multifractality, D=0.55. The figure also shows that the series has a high fractal asymmetry, A=0.42 (Figure 2E). The long right tail of the singularity spectrum indicates that the multifractal structure of the data series is less sensitive to local fluctuations of large magnitudes. Conversely, if a singularity spectrum has a long left tail, this means that its multifractal structure is less affected by local fluctuations with small magnitudes (see Ihlen, 2012). Figure 2F presents the singularity spectrum for *The Rainbow* by D. H. Lawrence with D=0.27 and A=-0.01. The values shown here illustrate that the series of the text has a degree of multifractality smaller than that of *Jane Eyre*, but it is (almost) symmetrical.

## 4. The JEFP Corpus

As mentioned in section 1, our corpus consists of three sub-corpora representing three major text categories: a collection of canonical fictional texts, a corpus of non-canonical fictional texts, and a corpus of non-fictional (expository) texts.

The canonical fictional sub-corpus comprises 77 English prose texts, written by 31 different authors, from Period C (1832–1900) and Period D (20th century) of the *Corpus of Canonical Western Literature* (Green, 2017)^7^. We selected those texts from the corpus that were sufficiently long for our analysis (at least 35K words).

The non-canonical fictional texts were downloaded from e-book publishing sites in the internet. We primarily used www.smashwords.com, an e-book distributor website that is catering to classic texts, independent authors and small press. It offers a large selection of books from several genres and allows downloads in various formats. The books are classified into “Fiction,” “Non-fiction,” “Essays,” “Poetry,” and “Screenplays.” We selected random books from various prose genres, using the site's filter to make sure that the books had a minimal length comparable to that of canonical texts.

We further supplemented the corpus of non-canonical books with the lowest rated books on www.goodreads.com and www.feedbooks.com, as well as books with the lowest rates of downloads on the Project Gutenberg site. These books are in the public domain, written mostly between 1880 and 1930 and more than 45K words in length. In this way, we obtained 95 books of non-canonical literature (from as many authors in each case). We made sure to collect non-canonical texts from the same time period as for our canonical sub-corpus to minimize the effect of phenomena, such as short-term language change on our analyses. However, collecting “low-quality” non-canonical texts from one century back is not easy, as texts of this category are unlikely to be preserved or even digitized. Those texts that survived are likely of relatively high quality. Therefore, our non-canonical sub-corpus can be regarded as a top-notch non-canonical, and thus, comparatively close to the canonical sub-corpus, which renders the classification tasks more difficult (section 5.5). Nevertheless, the non-canonical texts selected by us are clearly non-canonical in the sense that they currently do not belong to any canon of literature like the one that we used for the selection of canonical texts (Green, 2017).

**Figure 3:**
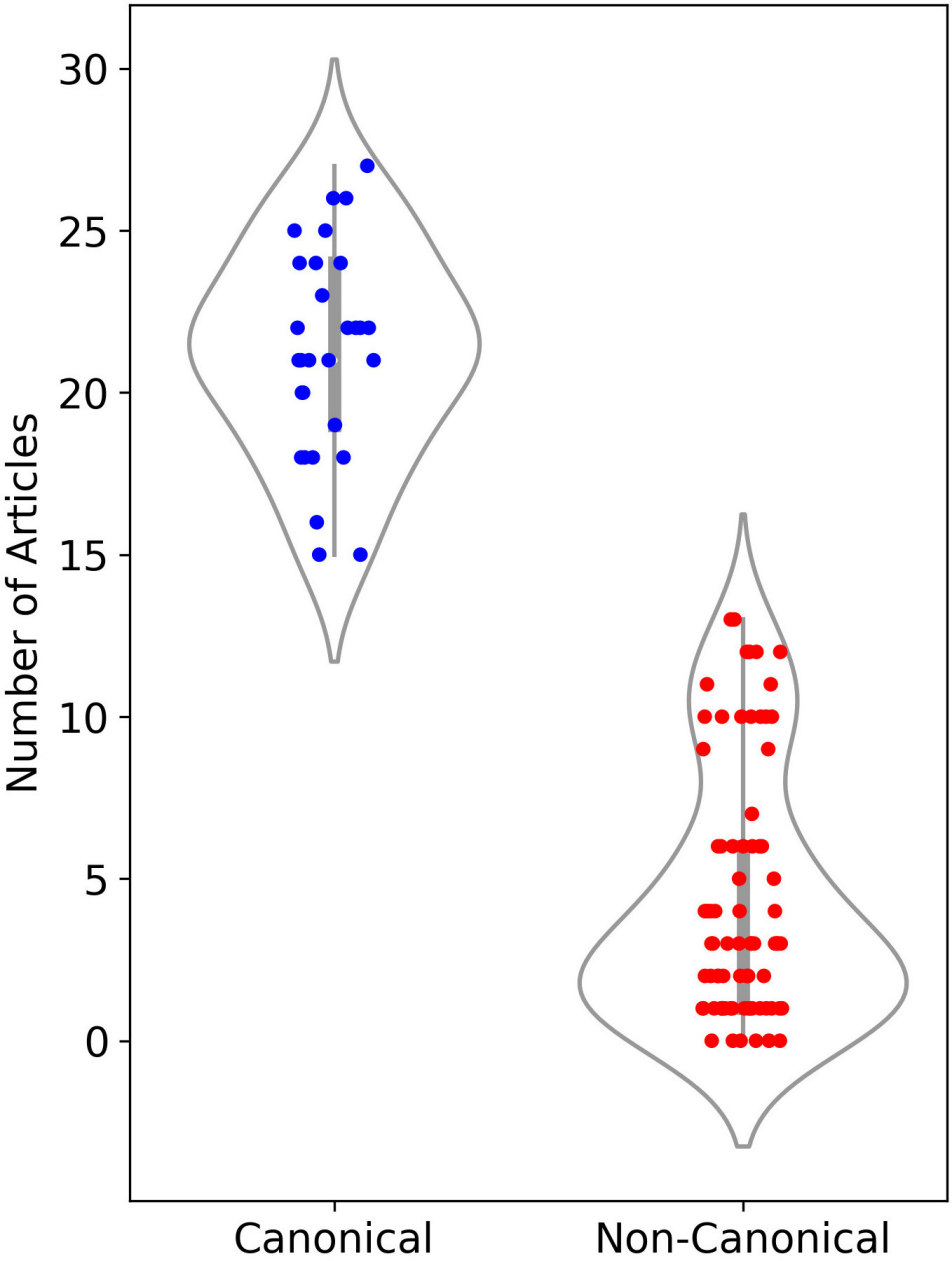
Number of articles in the top 30 language editions of Wikipedia for authors in the canonical (blue) and non-canonical (red) sub-corpora.

As another discriminating factor between canonical and non-canonical texts, we counted the number of articles that each author has in the top 30 language editions of Wikipedia. This measure is evidence for the international reputation of an author. Figure 3 shows a strip plot for all authors in each category. There is a clear separation between the authors of the two groups. All authors of canonical texts have at least 15 articles each in the 30 Wikipedia editions. In the non-canonical category, each author has up to 13 articles at most; for the majority of authors, the number is <5. These numbers provide independent evidence for the higher degree of prestige (Underwood and Sellers, 2016) of canonical authors, in comparison to non-canonical authors.

To compile the non-fictional sub-corpus we relied on Project Gutenberg. We downloaded all non-fictional books and randomly selected 132 books from different genres, such as architecture, astronomy, geology, geography, philosophy, psychology, and sociology. To increase the diversity, we added the first two volumes of *The Encyclopedia Britannica* published by the University of Cambridge and a text called *Glossary of Chess Terms* by Gregory Zorzos. This text was added to our corpus because of its extreme fractal behavior, as discussed in the previous section and shown in Figure 1. The texts of the two fictional categories, with the exception of the last one, were published in similar time periods.

Table 1 contains aggregate information about the length and time of publication of the texts contained in all categories. Information about the entire JEFP Corpus is provided in Supplementary Table S1. The mean lengths of the texts are different for each of the three text categories. It is important to mention that the exact length of a text does not affect the results of our experiments, given that the texts are sufficiently long to be analyzed robustly for their variability and fractal properties (see section 5.2). As far as the year of publication is concerned, the canonical fictional texts span a broader time period than the non-canonical texts. This is not surprising, as canonical literature represents a small selection of texts of a period, and thus constitutes a smaller population per time unit than non-canonical texts. In terms of both language history and literature periodization these differences are negligible.

**Table 1:** Number of texts, number of authors, mean text length (number of tokens), and mean year of publication (±SD) for the different text categories of the JEFP Corpus.

	**# Texts**	**# Authors**	**Length (**×10^3^**)**	**Year of publication**
Canonical	77	31	196 ± 91	1,870 ± 31
Non-canonical	95	80	102 ± 44	1,905 ± 19
Non-fictional	135	131	168 ± 193	1,902 ± 19

The texts were tagged manually to eliminate material not belonging to the core text, such as tables of contents and indices. Headers were left in the text, as they are potentially informative. Moreover, the texts were cleaned up semi-automatically using regular expressions to identify (and re-join) hyphenated words at the end of a line.

## 5. Analysis and Classification Results

The core hypothesis behind the present study is that the three different text categories under analysis—non-fictional texts, fictional/canonical and fictional/non-canonical ones—differ in terms of fractality and variability. The JEFP Corpus allows us to test this hypothesis, as it contains samples of text from the three categories of interest. In order to compare the text categories, we carried out bivariate as well as multivariate analyses. In the bivariate analyses we compare the various statistics across the three categories of text; in order to get an understanding of the interplay between, and relative importance of, the various features, we carried out two binary classification tasks. The first task (Task 1) is to separate the fictional from the non-fictional works. The second task (Task 2) consists in separating the canonical fictional texts from the non-canonical ones.

The series analyzed were derived from the four textual properties described in section 2, POS-tag frequencies, sentence length, lexical diversity, and topic distributions. The first two properties are regarded as correlates of lower-level processing (decoding) while the latter two are taken to correspond to higher-level processing (integration, see sections 1 and 2). In section 5.1 we describe how the basic measurements for these properties were obtained, converting the texts to series. Following some remarks concerning the validation of the methods (section 5.2) we present the results in section 5.3. In section 5.4 the source of the multifractality is discussed before we present the results of the classification tasks in section 5.5.

### 5.1. Converting Texts Into Series

To convert a text into a series of POS-tag frequencies, we determined the number of each specific tag in the sentences of the text. In our analysis, we focused on the major parts of speech, i.e., nouns, adjectives, verbs, and pronouns. For the annotations we used the Stanford POS-tagger (Toutanova et al., 2003). For the calculations, we included all types of nouns, i.e., singular as well as plural nouns and proper names. Several types of verb forms, e.g., base forms, past tense forms, gerunds, past participles—were all treated as verbs. The category of “adjective” includes simple, comparative as well as superlative adjectives. Pronouns are either personal or possessive. We thus obtained four different series derived from POS-tags.

Sentence length was measured in terms of tokens as delivered by the tokenizers of the NLTK-package for Python (Bird et al., 2009). The texts were first sentence-tokenized (split into sentences), and then each sentence was word-tokenized. The length of each sentence is the number of its tokens. A token is an instance of a word, number or punctuation mark in a text. Punctuation marks were not removed and treated as tokens.

Lexical diversity measures the richness of vocabulary of a text. Several metrics have been proposed for measuring lexical diversity. Type-Token Ratio (TTR) is the simplest one, in which the number of distinct words (types) is divided by the length of the text. However, TTR is highly sensitive to text length. In our experiments (cf. section 5), we therefore use the Measure for Textual Lexical Diversity (MTLD; McCarthy and Jarvis, 2010), which is more robust because it is less sensitive to text length. To convert a text into a series of lexical diversity values, we first segmented the text into segments of 100 tokens, which seemed like a good compromise between reliability of the calculations, and the required minimal length of series for fractal analysis. We then computed MTLD values for each segment to obtain a series for this feature.

Topic modeling is a high-level analysis of text that focuses on the content conveyed. To extract the topic distribution of a text, we first segmented the text into coherent chunks using the TopicTiling algorithm (Riedl and Biemann, 2012). Then, we applied Latent Dirichlet Allocation (LDA) (Blei et al., 2003; Griffiths and Steyvers, 2004) to all chunks of all texts in the corpus, thus obtaining a topic model. The number of topics, one of the hyperparameters of LDA, was set to 100. The resulting topic model is a statistical model that shows the importance of each word in a topic. Afterwards, the topic model was applied to each chunk of a text to infer the distribution of the 100 topics (the ‘topic probabilities’). In order to convert the vector of topic probabilities to a series, we calculated the Jensen–Shannon divergence of the topic representations of adjacent chunks.

### 5.2. Methodological Validation of Fractal Analysis of the Corpus

As the length of texts varies considerably in our corpus, we conducted an experiment to see whether text length affects the degree of fractality. For the text in the three categories, we chose the maximum scale, i.e., the maximum size of the windows, in such a way that the average of the maximum scales was similar for the three text categories. A statistical test showed no significant difference. Therefore, in our experiments we do not impose any restriction on the maximum scale. In the MFDFA method the scaling behavior of the fluctuation function, *F*_*q*_(*s*), is determined vs. the window size, *s*, i.e., $F_{q}\left(s\right)\sim s^{h\left(q\right)}$. By fitting lines to the double-log diagrams of the fluctuation function, *h*(*q*) is computed for different values of *q* and fractal features are then obtained. Looking at the linear fits and how well they have been fitted to the values reveals information about fractal regimes for different values of *q* in the text properties and for the three text categories. *R*^2^ is a statistical measure that determines how well a linear fit represents the data. We computed mean *R*^2^ values for each text category. For all values of *q* and for all text properties *R*^2^ is larger than 0.94, which means that linear fits are very precise and close to the observed values. The *R*^2^ values are summarized in Supplementary Figure S1.

### 5.3. Analysis of Variance and Fractality

After generating the series for the seven text properties for all texts, we calculated the variance, V, as a measure of how variable each text property was across each text. Moreover, we used MFDFA to calculate the following fractal features for each text: the degree of fractality (H), the degree of multifractality (D) and the degree of fractal asymmetry (A) (see section 3). As Kolmogorov-Smirnov tests revealed that some of the data were not normally distributed, the data was entered into a Wilcoxon test to assess the differences between the three sub-corpora, supplemented by non-parametric Mann-Whitney tests for all (*post-hoc*) pairwise comparisons. The median values of the variances and fractal features are shown in Table 2 for all three subcorpora of text (canonical, non-canonical, and non-fictional). In addition, we obtained the same statistics for both types of fictional text (canonical and non-canonical texts) together, as we distinguish two classification tasks: the distinction between fictional vs. non-fictional texts (Task 1), and between canonical vs. non-canonical texts (Task 2; see section 4).

**Table 2:** Median values of all text properties.

		**Noun**	**Verb**	**Adjective**	**Pronoun**	**Sentence length**	**MTLD**	**Topic distribution**
V	Lit.	11 (10, 13)	6.5 (5.6, 7.2)	2.3 (2.1, 2.8)	3.3 (3.0, 3.6)	220 (184, 277)	376 (361, 391)	4.5e-3 (4.4e-3, 4.6e-3)
	Non-Lit.	19 (17, 20)	7.5 (7.0, 8.3)	4.2 (3.9, 4.5)	1.9 (1.4, 2.1)	305 (290, 336)	322 (295, 348)	4.8e-3 (4.6e-3, 5.3e-3)
		***	**	***	***	***	***	***
	Can.	15 (14, 17)^b^	9.0 (7.2, 9.9)	3.3 (3.0, 3.9)^b^	4.3 (3.7, 5.0)^c^	321 (296, 367)	390 (375, 408)^c^	4.8e-3 (4.5e-3, 4.9e-3)
	Non-Can.	9.1 (8.1, 10)^c^	5.0 (4.5, 6.0)^c^	1.9 (1.7, 2.1)^c^	2.7 (2.4, 3.0)^c^	163 (145, 194)^c^	357 (345, 381)^b^	4.2e-3 (4.0e-3, 4.5e-3)^c^
		***	***	***	***	***	**	***
H	Lit.	0.714 (0.706, 0.725)	0.66 (0.65, 0.67)	0.685 (0.677, 0.695)	0.67 (0.66, 0.68)	0.70 (0.69, 0.71)	0.65 (0.64, 0.66)	0.63 (0.62, 0.65)
	Non-Lit.	0.69 (0.67, 0.71)	0.72 (0.70, 0.76)	0.72 (0.70, 0.74)	0.71 (0.70, 0.73)	0.73 (0.70, 0.75)	0.68 (0.66, 0.70)	0.66 (0.61, 0.69)
		*	***	***	***		***	
	Can.	0.72 (0.70, 0.73)	0.67 (0.65, 0.68)^c^	0.69 (0.68, 0.70)^a^	0.67 (0.65, 0.68)^c^	0.70 (0.68, 0.71)	0.64 (0.63, 0.66)^a^	0.64 (0.61, 0.66)
	Non-Can.	0.71 (0.70, 0.73)	0.66 (0.64, 0.67)^c^	0.68 (0.67, 0.69)^b^	0.67 (0.65, 0.68)^c^	0.70 (0.68, 0.71)	0.65 (0.64, 0.66)^b^	0.63 (0.61, 0.65)
D	Lit.	0.30 (0.26, 0.32)	0.20 (0.17, 0.21)	0.31 (0.28, 0.33)	0.67 (0.66, 0.68)	0.26 (0.24, 0.28)	0.20 (0.17, 0.22)	0.19 (0.18, 0.21)
	Non-Lit.	0.26 (0.23, 0.31)	0.37 (0.34, 0.40)	0.30 (0.25, 0.37)	0.71 (0.70, 0.73)	0.34 (0.29, 0.42)	0.20 (0.18, 0.22)	0.25 (0.20, 0.28)
			***		***	***		
	Can.	0.34 (0.32, 0.36)	0.23 (0.19, 0.25)^c^	0.32 (0.28, 0.34)	0.67 (0.65, 0.68)^c^	0.31 (0.26, 0.35)	0.20 (0.16, 0.22)	0.18 (0.15, 0.21)^a^
	Non-Can.	0.26 (0.23, 0.30)	0.16 (0.15, 0.20)^c^	0.29 (0.27, 0.33)	0.67 (0.65, 0.68)^c^	0.23 (0.20, 0.25)^c^	0.21 (0.17, 0.23)	0.20 (0.18, 0.21)
		***	***			***		
A	Lit.	0.03 (−0.05, 0.09)	0.09 (0.02, 0.14)	0.04 (−0.03, 0.07)	0.09 (−0.01, 0.17)	0.08 (0.01, 0.14)	0.11 (0.06, 0.18)	0.15 (0.06, 0.23)
	Non-Lit.	0.24 (0.12, 0.41)	0.61 (0.55, 0.68)	0.55 (0.48, 0.66)	0.36 (0.28, 0.48)	0.56 (0.45, 0.69)	0.15 (0.04, 0.28)	0.09 (−0.04, 0.27)
		***	***	***	***	***		
	Can.	0.09 (0.00, 0.13)^a^	0.10 (0.04, 0.16)^c^	0.04 (−0.03, 0.08)^c^	0.16 (0.05, 0.21)^b^	0.13 (0.04, 0.23)^c^	0.10 (0.03, 0.20)	0.20 (−0.02, 0.27)
	Non-Can.	−0.04 (−0.13, 0.06)^c^	0.07 (−0.03, 0.20)^c^	0.04 (−0.07, 0.11)^c^	−0.01 (−0.07, 0.20)^c^	0.02 (−0.02, 0.12)^c^	0.12 (−0.06, 0.24)	0.15 (0.05, 0.26)

The 95% confidence intervals are shown in parentheses. The rows represent the features analyzed (variance [V], degree of fractality [H], degree of multifractality [D] and fractal asymmetry [A]). Each feature is analyzed for two tasks: Task 1, fictional (Lit.; N = 172) vs. non-fictional (Non-Lit.; N = 135) texts, and Task 2, canonical (Can.; N = 77) vs. non-canonical (Non-Can.; N = 95) texts. The asterisks indicate whether the differences between the two text categories of a given task are statistically significant (Mann-Whitney test;

* *p ≤ 0.05*;

** p ≤ 0.01; and

*** *p ≤ 0.001). In addition, canonical and non-canonical texts are compared separately to non-fictional texts; the superscript numbers indicate significances (Mann-Whitney test; ^a^p ≤ 0.05; ^b^p ≤ 0.01; and ^c^p ≤ 0.001)*.

Table 2 shows that none of the text properties (four types of POS-tag frequencies, sentence length, lexical diversity and topic probabilities) results in significantly different median values for all features (variance and fractality measures) in both tasks. The higher-level properties (MTLD and topic distributions) do not vary significantly across text types for the fractal features. However, the variance (V) is significantly different for all features in both tasks. Strikingly, V values are always higher for non-fictional texts than for fictional texts, except for the values obtained from frequencies of pronouns, and from MTLD values. This difference is mainly driven by non-canonical fictional texts. V values for canonical texts range in between those for non-fictional and non-canonical texts. In some cases (verb frequencies, sentence length and topic distributions), the values for canonical texts are not significantly different from those of non-fictional texts, but higher than the values for non-canonical texts.

**Figure 4:**
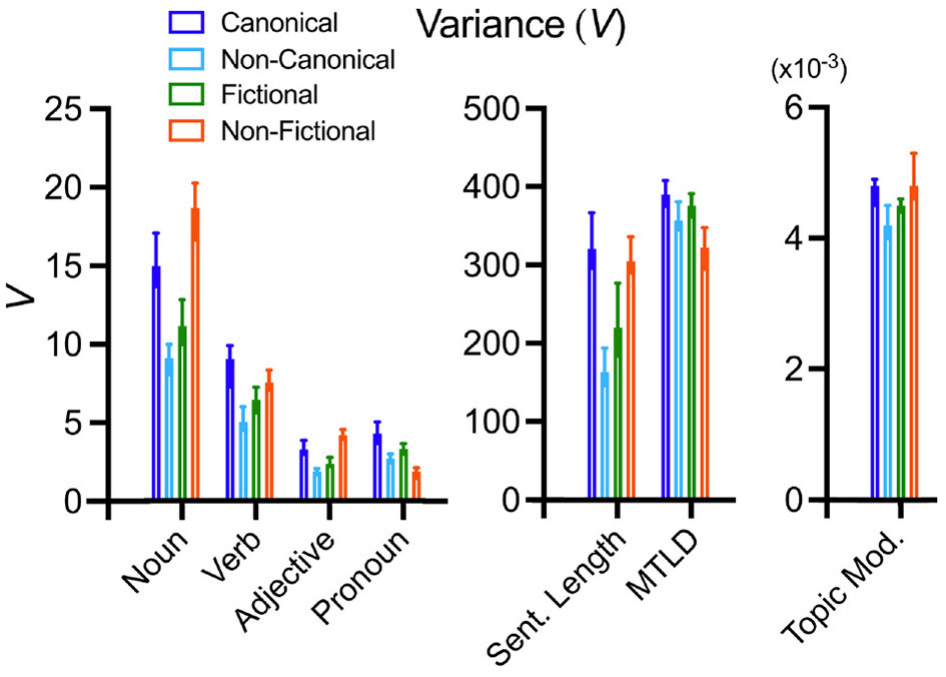
Plots of the median variances for all text properties. The colors indicate the different text categories, as indicated at the upper left-hand side of the figure. The whiskers indicate 95% confidence intervals. For significance levels of the differences, see Table 2. Sent. Length, sentence length; Topic Mod., topic modeling.

In summary, in terms of V, canonical texts are more similar to non-fictional texts than to non-canonical texts. Only for the frequency distribution of pronouns and MTLD values do the canonical texts exhibit the highest values, followed by non-canonical texts and, with even lower values, by non-fictional texts. Figure 4 shows the differences between the variances of the text categories for all properties. Note that the magnitude of the variances does not reflect the magnitude of the mean values for the text properties (cf. Supplementary Table S2 for the mean values).

**Figure 5:**
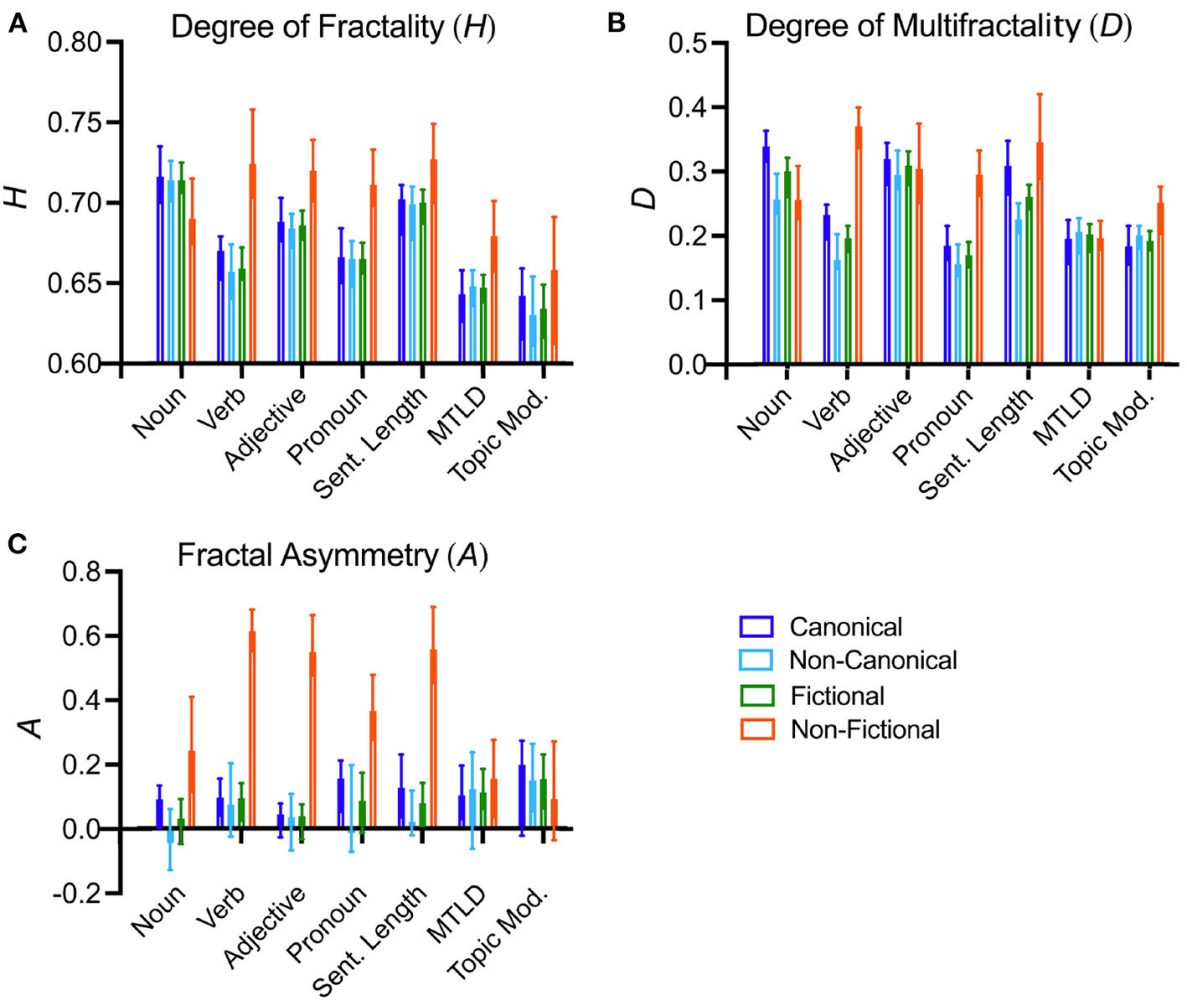
Plots of the median values for the degree of fractality **(A)**, the degree of multifractality **(B)**, and fractal asymmetry **(C)** for all text properties. The colors indicate the different text categories, as indicated on the down right of the figure. The whiskers indicate 95% confidence intervals. For significances of the differences, see Table 2. Sent. Length, sentence length; Topic Mod., topic modeling.

Results for the degree of fractality (H) are listed in Table 2 and the means are visualized in Figure 5A. The degree of fractality is of similar magnitude (closer to 0.5) for all text properties for canonical and non-canonical fictional texts. By contrast, the H values for non-fictional texts are generally higher than for either type of fictional text (canonical or non-canonical), with the exception of the frequencies of nouns, sentence length and topic distributions. These results suggest that a lower degree of long-range correlations might be a uniform characteristic of fictional texts as opposed to non-fictional texts, regardless of the status of the fictional texts as canonical or non-canonical.

As Table 2 and Figure 5B show, the values for the degree of multifractality, D, are significantly higher for the frequencies of verbs and pronouns as well as sentence length in non-fictional as opposed to fictional texts. A comparison of canonical and non-canonical fictional texts reveals that the D values of canonical texts are consistently higher than or equal to the values for non-canonical texts, even though this tendency reaches statistical significance only for the frequencies of nouns and verbs, as well as sentence length.

The degree of asymmetry, A, does not differ between canonical and non-canonical fictional texts (Table 2 and Figure 5C). For lower-level properties, fictional texts are rather symmetrical (i.e., A is close to 0), and A is much higher for non-fictional texts than for fictional texts. For the higher-level properties (MTLD values and topic distributions), A values do not vary across the three sub-corpora.

To summarize the observations made above, canonical fictional texts show more variability with respect to the properties measured in our study than non-canonical texts, and are, in this respect, more similar to non-fictional texts. However, the lower degree of fractality (H) suggests that the two types of fictional texts display a lower degree of long-range correlations than non-fictional texts do. Moreover, canonical texts tend to be more multifractal than non-canonical texts in terms of the frequencies of nouns and verbs, as well as for sentence length (higher D). Unlike in the case of non-fictional texts, the fractal spectra of fictional texts are rather symmetrical (A is closer to 0).

**Figure 6:**
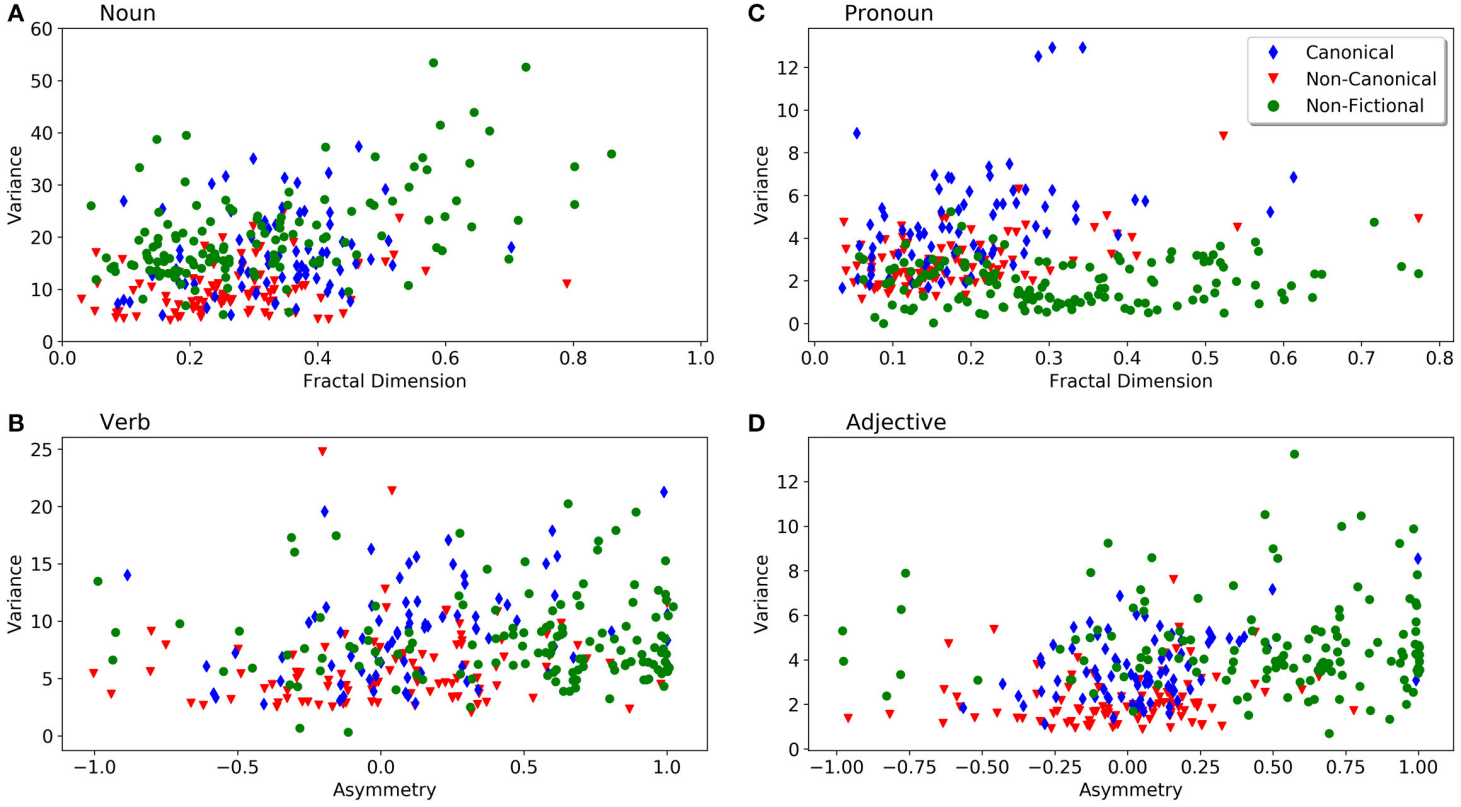
Scatter plots of variance (y-axis) and fractal features of POS-tags (x-axis). **(A)** Degree of fractality (H) and the variance of noun series. **(B)** Degree of multifractality (D) and the variance of verb series. **(C)** Degree of multifractality (D) and the variance of pronoun series. **(D)** Fractal asymmetry (A) and the variance of adjective series. Each dot represents one text from our corpus. For color coding of the text categories, see insert in **(B)**.

The individual values for the variance (y-axis) and fractal features (x-axis) for selected text properties are visualized as scatter plots in Figure 6 to illustrate the separation and overlap between the different text categories. For this figure, we chose plots that showed a relatively clear separation of the text categories by subjective visual inspection. Figure 6A shows the degree of multifractality and the variance of noun series. As stated above (Table 2), the variances for non-canonical texts tend to be lower than those of the other two categories. Figure 6B depicts the degree of multifractality and the variance of pronoun frequencies; it shows that fictional texts tend to have a higher variance compared to non-fictional texts. Both Figures 6A,B confirm that non-fictional texts scatter in a wider range of the degree of multifractality. In Figures 6C,D, the variances of verb and adjective series are plotted as a function of the degree of asymmetry. Fractal patterns of non-fictional texts are more asymmetrical (higher A). Again, canonical fictional texts exhibit a wider scatter, as variance is higher compared to non-canonical texts, which suggests a more diverse usage of language structures in the former category. The behavior of non-fictional texts varies across the tags. For example, the texts scatter more widely in the plot of adjectives (Figure 6C), while their pronoun variances cover a narrower range (Figure 6D), since pronouns are not so frequent in non-fictional texts (Supplementary Table S2). Figure 6 also illustrates that non-fictional texts have more complex fractal patterns and spread more broadly along the fractal feature (x-)axes. Non-fictional texts tend to show a higher fractal degree and more fractal asymmetry than fictional texts.

### 5.4. The Source of Multifractality

Both fictional and non-fictional texts are multifractal up to a certain degree, as can be seen from Table 2. It is therefore important to analyze the source of multifractality in the texts of the corpus. Multifractality in a series can be caused by (i) the presence of long-range correlations of small and large fluctuations, or (ii) a broad probability distribution (Kantelhardt, 2011). Therefore, we used the Iterative Amplitude Adjusted Fourier Transform (IAFFT) surrogate test to investigate the source of multifractality in the text property series. IAAFT retains the distribution and linear structures of series while destroying non-linear correlations. If the multifractality of a series is not due to non-linear correlations, IAAFT has no effect on the multifractality (for a comprehensive discussion on surrogate methods, see Lancaster et al., 2018).

We applied IAAFT surrogate tests to the series derived from all text properties, for all books in our corpus. We allowed the IAAFT algorithm to iterate up to 500 times to generate a surrogate. For each series we generated an ensemble of 100 surrogates and compared the degrees of multifractality of the series with those of the surrogates. For all texts in canonical, non-canonical and non-fictional categories as well as all 307 books in the corpus, we computed the percentage of the texts whose degree of multifractality is significantly larger than the mean degree of multifractality of the surrogates (*p* <0.05).

Before summarizing the results it is important to note that we do not expect all texts to exhibit multifractality. Our hypothesis says that texts from different categories may differ in terms of their degrees of multifractality. This implies that some texts will be more multifractal than others, and in fact, some texts are expected not to be multifractal at all. Nonetheless, we want to compare the results summarized in Table 2 with the results of the surrogate tests. We only provide a rough summary here. The results of the IAAFT surrogate tests are summarized in Supplementary Figure S2.

The results show that across the three text categories, more than 90% of all texts have a significantly higher degree of multifractality than their surrogates (on average), for all text properties. The only text property for which the average number is lower is topic distribution, with a value of 88%. For lower-level text properties and MTLD, more than 90% of texts have a higher degree of multifractality than their surrogates in all text categories, with the exception of sentence length in the non-canonical texts. More than 88% of the sentence-length series derived from the non-canonical texts and the topic distribution series derived from the fictional/non-canonical and non-fictional texts show a significant difference from their surrogates. The lowest value is the one for the topic distribution series of canonical literary texts (83%). We will see below (section 5.5) that in fact, the fractal features of topic distribution cannot classify the text categories with a high accuracy, either.

While we cannot offer a detailed assessment of surrogate analyses for all individual texts and all individual features, from our point of view the aggregate results make it very unlikely that observed instances of multifractality are not due to long-range correlations, though we cannot, of course, exclude that in individual cases they are caused by a broad probability distribution.

### 5.5. Classification

While a statistical analysis of features gives insights into the distribution of a single feature (cf. section 5.3), classification separates classes from each other, potentially in a non-linear fashion, which is a better way to detect differences between the text categories than a linear analysis of single properties. In this section, we describe the results for the classification of the text categories. As mentioned before, we distinguish two classification tasks: fictional texts are classified against non-fictional texts (Task 1), and canonical fictional texts against non-canonical fictional texts (Task 2).

For a better understanding of the postulated level of text processing, we present results for the lower-level and higher-level properties separately as well as in combination (Table 3). For classification, we used a Support Vector Machine (SVM) with a Radial Basis Function (RBF) kernel. As the features have varying scales, we normalized them to a mean of 0 and standard deviation of 1. The evaluation measure is balanced accuracy, which is a weighted average accuracy value that is proportional to the size of each class, and therefore, does not favor larger classes. We assessed the statistical significance of differences between settings by using a 5 ×2 cv paired *t* test (Dietterich, 1998) (significance level at *p* ≤ 0.05). In this test, 2-fold cross validation is repeated 5 times and the dataset is shuffled each time.

**Table 3:** Accuracy of classification (in %) for the non-fictional/fictional distinction (Task 1) and the canonical/non-canonical distinction (Task 2).

	**Task 1**		**Task 2**	
	**Variability**	**Fractal features**	**Variability**	**Fractal features**
Noun	71.0 ± 2.5	75.3 ± 2.3	69.5 ± 3.8	62.4 ± 3.0
Verb	56.8 ± 3.7	75.1 ± 1.5	68.3 ± 2.1	55.5 ± 3.0
Adjective	74.1 ± 2.7	80.4 ± 2.3	69.7 ± 4.0	51.6 ± 3.7^†^
Pronoun	69.5 ± 0.9	72.1 ± 1.8	68.0 ± 1.9	52.2 ± 4.7^†^
Sentence-length	65.0 ± 2.2	74.0 ± 2.0	69.3 ± 2.9	59.7 ± 3.2
MTLD	63.7 ± 2.3	56.9 ± 3.2	52.3 ± 3.3^†^	55.5 ± 3.1
Topic distribution	62.8 ± 2.3	64.0 ± 3.3	60.6 ± 3.4	49.2 ± 3.5^†^
Lower-level	92.4 ± 2.1	86.0 ± 2.0	71.6 ± 2.6	62.9 ± 3.9
Lower-level, Combined	94.9 ± 1.0		71.4 ± 4.8	
Higher-level	72.4 ± 1.9	63.2 ± 3.3	63.5 ± 3.2	57.1 ± 1.8
Higher-level, Combined	71.8 ± 2.9		61.9 ± 4.5	
Lower- & Higher-level	93.6 ± 1.3	84.9 ± 1.6	73.6 ± 2.3	65.0 ± 1.7
Lower- & Higher-level, Combined	94.7 ± 1.3		71.6 ± 3.6	

*Means±SD are listed (N = 10). All values are significantly different (p ≤ 0.05) from random accuracy (50%), except where indicated by a dagger (†)*.

Before we present our results, it is important to note that the objective of the classification task is not to obtain a maximum degree of accuracy in absolute terms. We are interested in a comparison of the relative discriminatory power of statistics capturing specific global structural properties (variability, [multi]fractality) obtained from specific observables (four types of POS-tags, sentence length, lexical diversity and topic distributions). While in computational linguistics it is customary to compare classification models to alternative models classifying the same textual material, such a comparison does not seem very informative to us for the purpose of our specific research question. It is to be expected that state-of-the-art language models, such as BERT (Devlin et al., 2019), will achieve much higher classification results than any of the models trained by us. In fact, Louwerse et al. (2008) already achieved 100% accuracy of text classification with a bigram model distinguishing “Literature” from “Non-literature.” We use the classification procedure as a way of understanding the relationship between the various predictor variables, i.e., as a tool for multivariate analysis, in an empirical study motivated by theoretical research questions. Note also that even in absolute terms, a comparison with other models would only make sense if the models used a comparable number of features. Readers interested in accuracy scores obtained in the classification of fictional/literary texts are referred to van Cranenburgh and Bod (2017) (see for instance the table on p. 1234).

In Table 3, we report the mean and the standard deviation for the 10 runs for each setting. The top part of Table 3 shows the classification results for individual properties. The analysis of variability provides comparable accuracies in Task 1 and Task 2. Exceptions are provided by verb frequency, which leads to much higher classification rates in Task 2 than in Task 1, and MTLD values, which are better predictors in Task 1. The best performance is observed for adjective frequency, which yields the highest accuracy of all predictors in Task 1, and which provides the best results in Task 2 as well (see also Table 2). The variance of MTLD values is more powerful in distinguishing fictional texts from non-fictional text (Task 1), but it cannot separate canonical from non-canonical texts in Task 2. As a lexical diversity measure, MTLD reflects the richness of vocabulary of a text. To get a better understanding of lexical diversity of fictional and non-fictional texts, we submitted the global MTLD-values of the texts, grouped into the categories “non-fictional,” “fictional/canonical,” and “fictional/non-canonical,” to an ANOVA. The test did not reveal a significant difference between the lexical diversity of the text categories (p=0.68). This finding is surprising, as lexical diversity is often regarded as a hallmark of good authorship, and can thus be expected to vary across the sub-corpora of interest.

The fractal features result in better accuracies in Task 1 than in Task 2 for all properties, with the exception of MTLD, which performs similarly in both tasks. The highest classification rate for Task 1 is, again, obtained for adjective series (80.4%). The series of lower-level properties, i.e., POS-tags frequencies and sentence length, perform well in Task 1. By contrast, the fractal features cannot distinguish well between canonical and non-canonical fictional texts (Task 2). This result is in accordance with the finding that the degree of fractality (H) and the degree of asymmetry (A) are of similar magnitude for canonical and non-canonical texts for almost all text properties (cf. Table 2).

The POS-tag frequencies and sentence length are regarded as lower-level properties and MTLD and topic distribution as higher-level properties. The top part of Table 3 presents the classification results for variance and the fractal features separately. When combining the two feature groups for all lower-level and all higher-level properties, as shown in the middle part of the table, a considerably improved accuracy is achieved in Task 1. Although the variance of each property alone does not provide a classification accuracy higher than 74% (for adjective frequencies), their combination effectively raises the accuracy up to 92%. Using all fractal features together for the classification task also increases the performance considerably. Finally, when all variances and fractal features are combined, the performance gets even better. A 5 ×2 cv paired *t* test confirms that all of these improvements are significant. In Task 2, we do not observe such a large improvement by accumulating the variances or the fractal features. For example, the performance of a model combining all variances of lower-level features is only slightly better than the performance of the variance of noun or adjective frequencies. For the fractal features, the classification accuracy of the combined model is similar to that of noun series only. The combination of all features does not offer any improvement either.

We also ran the classification task using all higher-level properties. In Task 1 (cf. the middle part of Table 3), the combination of the variances of two higher-level properties results in a considerable improvement. By contrast, a combination of the fractal features leads to no improvement. It is therefore expected that the combination of all variances and fractal features does not improve classification. Adding more features to an SVM classifier may actually decrease the classification result, because the SVM classifier tries to maximize generalization. Such a decrease is observed if all features are combined together. In Task 2, we can see that the combination of variances of the higher-level features improves the classification results, though not for the fractal features. The accumulation of all features does not provide any obvious improvement either.

Lower-level and higher-level properties can be combined to analyze the different classes of text, as shown at the bottom of Table 3. In Task 1, we observe no improvement when combining all variances or all fractal features. Finally, the result obtained by combining all features is not significantly different from the classifier that was trained on all features (variances and fractal features) of lower-level properties. In Task 2, when all variances or all fractal features are taken into account, an improvement can be observed. The combination of all features does not, however, improve the accuracy of the model compared to the model trained on all variances.

In summary, the results of the classification experiment show that lower-level properties are more effective in distinguishing fictional text from non-fictional text (Task 1) than higher-level properties. Even individual properties—the frequencies of nouns and verbs—reach accuracies higher than 70%, or even 80% in the case of the fractal features for adjectives. By combining lower-level features in the classification task, the accuracy reaches 95%. The accuracy values for Task 2 range between 68 and 70% for individual lower-level features, and are much lower for higher-level features. The performance of the classifier does not improve significantly if the lower-level features are combined, and the resulting accuracy score (71.6%) is not significantly higher than the score for adjective frequencies (69.7%). This finding points to a strong correlation of the lower-level features in Task 2^8^.

## 6. Discussion and Conclusions

The starting point of this article was the question of whether canonical and non-canonical fictional texts exhibit systematic differences in terms of structural design features. In order to put any observable differences into perspective, we also included non-fictional (expository) texts for comparison. Our study was inspired by findings from the field of vision, where aesthetic experience has been linked to the structural features of variability (measured in terms of variance) and fractality or self-similarity. As pointed out in section 1, the transfer from vision to reading has obvious limitations. Still, given the widespread assumption of domain-general processes in the processing of language (Diessel, 2019), we tested to what extent the features that have been observed to correlate with observers' preferences in vision differentiate canonical from non-canonical fictional texts, and fictional from non-fictional texts.

We used four features as the basic measurements, classified into lower-level features (frequencies of POS-tags and sentence length) and higher-level features (lexical diversity and topic distributions). The global structural design features that we investigated were those that have been shown to be prominent in vision (variability, fractality). By applying the relevant statistical methods to series derived from the four types of text properties we generated global statistics of various types. In our analysis we proceeded in two steps: First, we carried out bivariate comparisons between the three text categories under analysis, for each feature separately. Second, we used the features to classify the three text categories in question, thus determining the relative importance of each feature as well as their combined discriminatory power.

In what follows we discuss our findings and their implications with a focus on the central questions addressed in this article.

### 6.1. Lower-Level and Higher-Level Text Properties

Our results have shown that generally speaking, the lower-level properties from which we derived series are better discriminators than the higher-level features, for the three text categories of interest. The differences between the text categories are more pronounced in bivariate comparisons, and the accuracy levels reached in the classification tasks are significantly higher for lower-level properties than for higher-level properties. This finding has some parallels obtained in research on other sensory domains. In the visual domain, the global spatial distribution of several low-level properties (for example, luminance changes, edge orientations, curvilinear shape and color features; see section 1) has been related to the global structure of traditional artworks and other preferred visual stimuli. In the auditory domain, music has been shown to be characterized by fluctuations in low-level features, such as loudness and pitch (Voss and Clarke, 1975), frequency intervals (Hsü and Hsü, 1991), sound amplitude (Kello et al., 2017; Roeske et al., 2018), and other simple metrices, such as measures of pitch, duration, melodic intervals, and harmonic intervals (Manaris et al., 2005), as well as patterns of consonance (Wu et al., 2015). These and many other studies indicate that low-level properties of music show long-range correlations that are scale-invariant and obey a power law. Interestingly, similar results were obtained for animal songs (Kello et al., 2017; Roeske et al., 2018).

Why are lower-level text properties informative with respect to the three text categories under analysis? We surmise that lower-level properties of text to some extent reflect discourse modes (Smith, 2003). These modes—Narrative, Report, Description (temporal), Information and Argument (atemporal)—are associated with different frequency distributions of POS-tags (cf. also Biber, 1995, who uses more specific categories in his multi-dimensional register analysis, however). For example, the Narrative mode is associated with verbs, while Description requires more adjectives. In a comparison of fictional and non-fictional text, it is moreover important to bear in mind that fictional text implies both external communication (between the narrator and the reader) and internal communication (between the protagonists, in the form of dialogues) as well as internal monologs and thoughts. Our results suggest that non-fictional texts show more global variability between discourse modes than fictional texts. Canonical fictional texts seem to pattern with non-fictional texts in terms of their higher global variability, in comparison to non-canonical fiction. While this hypothesis requires more (qualitative as well as quantitative) in-depth studies, it suggests that canonical authors may use a richer variety of discourse modes (or narrative techniques) than non-canonical authors. We intend to test this hypothesis in future studies.

Considering the higher-level properties, only one of the four features studied, the variance V, showed differences between all of the three text categories. No significant differences were observed for any of the fractal features, with the exception of the Hurst exponent H) determined on the basis of MTLD measurements, which is higher for non-fictional than for fictional texts (cf. Table 2). Accordingly, the classification rates obtained by using higher-level features only are relatively low (up to 71.8% for the classification of fictional vs. non-fictional texts, and 61.9% for the classification of canonical fictional vs. non-canonical fictional texts; cf. Table 3). However, when comparing lower-level and higher-level properties and their distributions in different text types it should be borne in mind that higher-level properties, in particular thematic structure across a text, cannot easily be measured. We have used the distribution of topic probabilities across texts as an indicator of thematic organization. It is of course conceivable that this way of operationalizing thematic structure is imperfect, or at least does not measure properties that have correlates in reading comprehension. In future studies, we will therefore experiment with a broader range of properties, including measurements of cohesion like those provided by Coh-Metrix (Graesser and Kulikowich, 2011; McNamara and Graesser, 2020).

### 6.2. Variability and Fractality/Long-Range Correlations

In vision, variability and fractality have both been shown to be important discriminators of stimuli, correlating with observers' preferences (see section 1). Our results show variability of the text properties to discriminate better between the three text types under analysis than statistics derived from MFDFA (93.6 vs. 84.9% for fictional vs. non-fictional texts, and 73.6 vs. 65% for canonical vs. non-canonical fiction). This suggests that long-range correlations play a minor role in the distinction between the three text categories under study.

In general, the variability of canonical fictional texts is higher than the variability of non-canonical texts, for all properties investigated by us. The results concerning the variability of non-fictional texts in comparison to fictional texts are less clear. For most properties, variability is higher for non-fictional than for fictional texts. As a result, the variability of canonical fictional texts is closer to (or the same as) that of non-fictional texts. Only for pronoun frequencies and MTLD values can a different pattern be observed. Here, canonical texts are more variable than both non-canonical and non-fictional texts.

It may be surprising to find that canonical fictional texts are, in some respects, more similar to non-fictional texts than they are to non-canonical fictional texts. However, in studies on reading difficulty it has been found that “[n]arrative texts are more easily understood than expository texts,” and “read nearly twice as fast” (McNamara et al., 2013, p. 93). Canonical texts are often regarded as being more demanding than popular literature, and have often been written with a different purpose, and for a different readership (learned/educated readers). What McNamara et al. (2013, p. 93) write about expository texts—“they tend to include less familiar concepts and words and require more inferences on the part of the reader”—may apply to canonical fiction to a greater extent than it applies to non-canonical fiction.

Long-range correlations in general seem to be slightly more pronounced in non-fictional texts than in fictional texts. The Hurst exponent, H, for the frequencies of verbs, adjectives and pronouns (as well as for MTLD) is significantly higher for non-fictional texts than for fictional texts (Task 1), and non-fictional texts display higher degrees of fractal asymmetry than fictional text (Task 1), for all lower-level properties. The classification experiments, however, show that fractality features do not discriminate as well as variability features (86.0 vs. 92.4% for Task 1, and 62.9 vs. 71.6% for the lower-level features).

In the visual domain, traditional artworks can be characterized by an intermediate to high degree of self-similarity (Braun et al., 2013; Brachmann and Redies, 2017). In the Fourier domain, large subsets of traditional artworks have spectral properties similar to pink noise, with a power (1/*f*^*p*^) spectral exponent around *p* = 1 (Graham and Field, 2007; Redies et al., 2007), which is also characteristic of many (but not all) natural patterns and scenes (Tolhurst et al., 1992). In MFDFA, this corresponds to a Hurst exponent of 1, while H=0.5 indicates white noise (no long-range correlations, corresponding to a Fourier power spectral exponent of 0). The median H value for the different text properties ranges from 0.63 to 0.73 in our study, confirming previous results for sentence length by Drożdż et al. (2016). This degree of self-similarity thus lies in between that of most natural signals and random (white) noise. The relevance of this finding requires further exploration.

### 6.3. Outlook

This study has been exploratory in several respects. It is based on a limited selection of text properties (frequency distributions of POS-tags, sentence length, lexical diversity, topic distributions) whose use was motivated by general considerations and assumptions concerning language processing and comprehension (cf. section 2), with the intention of identifying those features that are potentially relevant to an understanding of the differences between canonical and non-canonical fiction in terms of global structural design features. There are, of course, many other text properties that are potentially relevant to our endeavor, e.g., those used for text assessment. In future studies, we intend to use a broader set of text properties from which we can derive series, specifically taking into account additional features reflecting cohesion (Graesser et al., 2003; McNamara et al., 2013).

Originally inspired by results from vision, our study has also shown that—valuable though this inspiration has been—there are a number of limitations to the analogy, and for the study of global text design a methodological toolbox of its own is needed. Given that reading has a temporal dimension, the question of predictability may play an important role. In section 2 we mentioned that language modeling could be used to statistically analyze a text. We intend to explore such methods in the future.

Another direction in which the research programme of empirical textual aesthetics should be extended concerns the textual material. We investigated English texts only, and these texts were taken from a restricted time period (19th and early 20th centuries). In order to see whether any of the present findings can be generalized to other types of fictional texts, other languages or other time periods would have to be investigated separately.

Finally, a major challenge for the future concerns the relationship between structure observed in series derived from texts on the one hand, and aesthetic experience on the other. By studying structural differences between text categories that reflect preferences of societies—canonical texts are “privileged” because they are attributed a high cultural value—we have taken a first step in this direction, but aesthetic experience itself can only be studied experimentally. Before experiments can be run, however, it will be necessary to gain a better understanding of the (measurable) text properties, and the types of patterns exhibited by these properties, that can reasonably be assumed to have behavioral or neural correlates. Further observational (corpus) studies, with extensions of the type pointed out above, are good way of gaining such insights.

## Data Availability Statement

The datasets presented in this article are not readily available because, due to copyright restrictions, the corpus cannot be made public. Information to download the texts in the corpus is provided in the Supplementary Table S1. Requests to access the datasets should be directed to mahdi.mohseni@uni-jena.de.

## Author Contributions

MM, VG, and CR developed the idea for the present work and wrote the manuscript. MM designed the experiments, wrote the code, carried out the experiments, and analyzed the data. VG and MM collected the datasets and prepared them for analysis. All authors contributed to the article and approved the submitted version.

## Conflict of Interest

The authors declare that the research was conducted in the absence of any commercial or financial relationships that could be construed as a potential conflict of interest.

## Footnotes

^1^The “classic” model—the LaBerge/Samuels model of automatic information processing in reading (cf. LaBerge and Samuels, 1974; Samuels, 1994)—assumes four components, (i) visual memory (VM), (ii) phonological memory (PM), (iii) semantic memory (SM), and (iv) episodic memory (EM). VM and PM are closely connected to sensory experience, i.e., visual and acoustic perception, and they are the input gates to processing in reading. Semantic memory is not only the place where “individual word meanings are produced,” but also “where the comprehension of written messages occurs” (Samuels, 1994, p. 710). It is thus also responsible for the linguistic process of decoding, including the processing of morphology (word structure) and syntax (sentence structure). Episodic memory is the place where propositional information is stored, and it is “responsible for putting a time, place and context tag on events and knowledge” (Samuels, 1994, p. 710).

^2^An exception is provided by McNerney et al., 2011, who had participants read a 361 pages long novel. For longer texts, human ratings have also been used as behavioral correlates of text structure (e.g., van Cranenburgh and Bod, 2017; van Cranenburgh et al., 2019). A methodological toolbox for measuring reading experience has been proposed by Knoop et al. (2016) and Thissen et al. (2018).

^3^Fractality has also been studied in reading, see Wallot et al. (2014).

^4^Language modeling is an essential part of many language processing tasks, such as machine translation, summarization and speech recognition. A language model computes the probability of a sequence of words and predicts the probability of the next word (Jurafsky and Martin, 2009). Language models capture both semantic and structural information, as the probability for a given word to occur is a function of both the surrounding structure and the semantic context.

^5^Embedding vectors—n-dimensional vectors of floats—represent the distribution of a linguistic segment and allow for the computation of (dis)similarities between segments. A wide variety of models have been proposed to represent text at the level of sub-word, word, sentence, etc. (for example, see Pennington et al., 2014; Bojanowski et al., 2017; Devlin et al., 2019).

^6^For example, Coh-Metrix measures complexity in terms of NP-density, the number of higher-level constituents and the presence of logical connectors, see Graesser et al. (2003).

^7^It is an interesting question, beyond the scope of this study, whether a different canon, e.g., a canon of African American Literature (cf. Gates and McKay, 2004)—would yield different results.

^8^Instead of the variance, which is the second moment of the sample data, one can run classification using the first moment, i.e., the mean. Although this is not the focus of our study, we also carried out the classification tasks using the means of the text properties. The results are provided in Supplementary Table S3. The results show that the performance of the models is slightly better using means rather than variances in distinguishing fictional from non-fictional texts (Task 1), but the variances provide better classification results in separation of the canonical from the non-canonical texts (Task 2), which is the more difficult task.
