# Supplementary material for: Fractality and Variability in Canonical and Non-Canonical English Fiction and in Non-Fictional Texts
Source: Front Psychol. 2021 Mar 31;12:599063. doi: 10.3389/fpsyg.2021.599063 (PMC8044424; doi:10.3389/fpsyg.2021.599063)
Supplement: Supplementary file 1 [file Data_Sheet_1.pdf]

Supplementary Material

ALTERNATIVE GLOBAL MEASURES OF VARIABILITY AND SELF-SIMILARITY

1 ENTROPY-BASED METHODS

Entropy, which is related to variability, measures uncertainty or (ir)regularity of a state or phenomenon represented by a random variable. If X is a discrete random variable with a set of possible values $\{x_1, x_2, \cdots, x_n\}$ and a corresponding probability function $P(X) = \{P(x_i), P(x_2), \cdots, P(x_n)\}$, the entropy of X is defined as:

$$H(X) = -\sum_{i=1}^{n} P(x_i) \log_b P(x_i)$$

Entropy is zero when the state is certain and it is highest when the all possibilities are equally likely to occur, i.e. when uncertainty is maximal. The basic formula of entropy or its extensions have been utilized for text analysis previously.

Rosso et al. (2009) applied statistical complexity and entropy quantifiers to a collection of poems and plays. Their analyses revealed that poems have a higher complexity than plays and Shakepeare's work is interestingly more homogeneous than that of his contemporaries and is exceptionally close to the average use of words in that time period. Chang et al. (2017) defined the information-based energy, combined from the relative temperature and information Shannon entropy, to quantify text complexity and an author's performance. Applying this method to texts of an English and an Chinese author, Shakespeare and Jin Yong, they showed that their more popular works have higher information-based energy. Hernández-Gómez et al. (2017) used an entropy-based method, called approximate entropy, to measure the degree of irregularity or randomness in a series. They applied this method to 14 different languages which belong to four linguistic families: Romance, Germanic, Slavic and Uralic. They showed that the languages exhibit different levels of irregularity which were similar for languages that belonged to the same family. The entropy of word distributions can also be informative for comparing different types of languages in term of word ordering. Montemurro and Zanette (2016) used entropy-based measures to show that word ordering is highly similar over several language families. Febres and Jaffe (2017) studied entropy and symbolic diversity of fictional texts of Nobel and non-Nobel laureates in English and Spanish. While they presented some results to show that there is a correlation between these global statistical properties and the quality of writing, they did not classify different groups of texts.

2 BOX COUNTING

There are several methods to measure fractality and the scaling behavior of structures. These methods typically represent measurements at different scales. Fractal analysis techniques have been widely applied to images (Wendt and Abry, 2007; Li et al., 2009; Wendt et al., 2009; Ji et al., 2013), including artworks (Taylor, 2002; Redies et al., 2007; Spehar et al., 2016). They are therefore of special interest for analyzing aesthetic phenomena.

One of the most widely used fractal analysis methods is box counting, which is mathematically straightforward and easy to apply. Given an object S, for a $\delta > 0$ the smallest possible number of

subsets with a diameter of at most δ, $N_\delta(S)$, which covers S, is found. For 1d objects, subsets are rulers and δ is their length. For 2d objects, subsets are boxes and δ is their area, and so forth. The growth ratio of $N_\delta(S)$, as $\delta \to 0$, reflects the degree of fractality of S. If $N_\delta(S)$ can be approximated by

$$N_\delta(S) \simeq c\delta^{D_B}$$

for a constant c, then D_B is called the box-counting dimension and shows how complex S is.

Mehri and Lashkari (2016) applied this method to seven famous text books and computed their degree of fractality by averaging the fractality degrees of word occurrences. The results revealed that all texts are fractal and their fractal dimensions differed slightly. Fractality patterns of series sometimes do not lend themselves to analysis with a single scaling measure. If different subsets of a series exhibit different types of scaling behavior, the series is multifractal. Chatzigeorgiou et al. (2017) used box counting to find the origin of multifractality in the word-length representation of texts in several Western languages. They showed that the long-range correlations in natural language are related to the clustering feature of long words, i.e. rare and often highly informative content words.

3 WAVELET-BASED METHODS

Fractal analysis methods based on wavelets are another family of techniques for studying scale-invariant properties of signals (Muzy et al., 1993; Wendt and Abry, 2007; Leonarduzzi et al., 2016). The wavelet transform (WT) is a method to analyze non-stationary signals. The WT of a signal X is defined as (Mallat, 1999):

$$T_\psi[X](a, t_0) = \frac{1}{a}\int_{-\infty}^{+\infty} X(t)\psi(\frac{t - t_0}{a})dt,$$

and it describes the content of X around a time parameter t_0 and a scale parameter a. ψ is the analyzing wavelet whose $n + 1$ first moments are zero, i.e. $\int_{\mathbb{R}} t^n \psi(t)dt = 0$, which makes the WT insensitive to possible polynomial trends of order n in the signal, something which is necessary for multifractal analysis (Muzy et al., 1994; Arneodo et al., 1995). The WT modulus maxima (WTMM) is a well-known method for analyzing multifractality and it is based on the WT coefficients. WTMM is defined by the local maxima $\mathcal{L}(a)$ of $|T_\psi[X](a, t)|$ according to a given scale a. Then the following partition function is defined:

$$Z(q, a) = \sum_{l \in \mathcal{L}(a)} |T_\psi[X](a, t)|^q \sim a^{\tau(q)}$$

If the signal is monofractal, $\tau(q)$ is independent of q. For multifractal signals, the scaling behavior cannot be explained with one value, so, $\tau(q)$ changes for different values of q. Based on WT and WTMM, other methods have been extended for discrete and multi-dimensional series (for example, see Wendt and Abry, 2007; Leonarduzzi et al., 2016). Although wavelet-based methods have been applied to a variety of fields, they have been rarely used in text processing. Leonarduzzi et al. (2017) applied the wavelet p-leader method to the sentence-length series of novels that were written either for young people or adults. The authors showed that the latter category is more diverse in terms of its degree of multifractality.

4 FRACTALITY AND CROSS-CORRELATION ANALYSIS

Fractal analysis can be extended to analyzing more than one series, in order to find relations between fractal behaviors of multiple series. Detrended Cross-Correlation Analysis (DCCA) (Podobnik and Stanley, 2008) and Multi-Fractal Detrended Cross-Correlation Analysis (MFDCCA) (Jiang and Zhou, 2011) are two methods for analyzing correlations between two series. Ghosh et al. (2019) applied MFDCCA, also known as MFDXA, to study correlations between two Tagore's poems, one written in Bengali and one in English. They found a nonlinear correlation between the poems. In a similar study, birdsong and human speech were compared by computing the mutual information decay of signals and it was concluded that the two vocal communication signals have similar dynamics (Sainburg et al., 2019).

REFERENCES

Arneodo, A., Bacry, E., and Muzy, J. (1995). The thermodynamics of fractals revisited with wavelets. *Physica A: Statistical Mechanics and Its Applications* 213, 232–275. doi:10.1016/0378-4371(94)00163-N

Chang, M.-C., Yang, A. C.-C., Stanley, H. E., and Peng, C.-K. (2017). Measuring information-based energy and temperature of literary texts. *Physica A: Statistical Mechanics and Its Applications* 468, 783–789. doi:10.1016/j.physa.2016.11.106

Chatzigeorgiou, M., Constantoudis, V., Diakonos, F., Karamanos, K., Papadimitriou, C., Kalimeri, M., et al. (2017). Multifractal correlations in natural language written texts: Effects of language family and long word statistics. *Physica A: Statistical Mechanics and Its Applications* 469, 173–182. doi:10.1016/j.physa.2016.11.028

Febres, G. and Jaffe, K. (2017). Quantifying structure differences in literature using symbolic diversity and entropy criteria. *Journal of Quantitative Linguistics* 24, 16–53. doi:10.1080/09296174.2016.1169847

Ghosh, D., Chakraborty, S., and Samanta, S. (2019). Study of translational effect in tagore's gitanjali using chaos based multifractal analysis technique. *Physica A: Statistical Mechanics and Its Applications* 523, 1343–1354. doi:10.1016/j.physa.2019.04.171

Hernández-Gómez, C., Basurto-Flores, R., Obregón-Quintana, B., and Guzmán-Vargas, L. (2017). Evaluating the irregularity of natural languages. *Entropy* 19, 521. doi:10.3390/e19100521

Ji, H., Yang, X., Ling, H., and Xu, Y. (2013). Wavelet domain multifractal analysis for static and dynamic texture classification. *IEEE Transactions on Image Processing* 22, 286–299. doi:10.1109/TIP.2012.2214040

Jiang, Z.-Q. and Zhou, W.-X. (2011). Multifractal detrending moving-average cross-correlation analysis. *Physical Review E* 84, 016106. doi:10.1103/PhysRevE.84.016106

Leonarduzzi, R., Abry, P., Jaffard, S., Wendt, H., Gournay, L., Kyriacopoulou, T., et al. (2017). P-leader multifractal analysis for text type identification. In *2017 IEEE International Conference on Acoustics, Speech and Signal Processing (ICASSP)*. 4661–4665. doi:10.1109/ICASSP.2017.7953040

Leonarduzzi, R., Wendt, H., Abry, P., Jaffard, S., Melot, C., Roux, S., et al. (2016). p-exponent and p-leaders, Part II: Multifractal analysis. Relations to detrended fluctuation analysis. *Physica A: Statistical Mechanics and Its Applications* 448, 319–339. doi:10.1016/j.physa.2015.12.035

Li, J., Du, Q., and Sun, C. (2009). An improved box-counting method for image fractal dimension estimation. *Pattern Recognition* 42, 2460–2469. doi:10.1016/j.patcog.2009.03.001

Mallat, S. (1999). *A Wavelet Tour of Signal Processing (2. ed.)*. (Cambridge: Academic Press)

Mehri, A. and Lashkari, S. M. (2016). Power-law regularities in human language. *The European Physical Journal B* 89, 241. doi:10.1140/epjb/e2016-70423-9

Montemurro, M. A. and Zanette, D. H. (2016). Complexity and universality in the long-range order of words. In *Creativity and Universality in Language*, eds. M. Degli Esposti, E. G. Altmann, and F. Pachet (Cham: Springer). 27–41. doi:10.1007/978-3-319-24403-7_3

Muzy, J.-F., Bacry, E., and Arneodo, A. (1993). Multifractal formalism for fractal signals: The structure-function approach versus the wavelet-transform modulus-maxima method. *Physical Review E* 47, 875–884. doi:10.1103/PhysRevE.47.875

Muzy, J.-F., Bacry, E., and Arneodo, A. (1994). The multifractal formalism revisited with wavelets. *International Journal of Bifurcation and Chaos* 4, 245–302. doi:10.1142/S0218127494000204

Podobnik, B. and Stanley, H. E. (2008). Detrended cross-correlation analysis: a new method for analyzing two nonstationary time series. *Physical Review Letters* 100, 084102. doi:10.1103/PhysRevLett.100.084102

Redies, C., Hasenstein, J., and Denzler, J. (2007). Fractal-like image statistics in visual art: similarity to natural scenes. *Spatial Vision* 21, 137–148. doi:10.1163/156856807782753921

Rosso, O. A., Craig, H., and Moscato, P. (2009). Shakespeare and other english renaissance authors as characterized by information theory complexity quantifiers. *Physica A: Statistical Mechanics and Its Applications* 388, 916–926. doi:10.1016/j.physa.2008.11.018

Sainburg, T., Theilman, B., Thielk, M., and Gentner, T. Q. (2019). Parallels in the sequential organization of birdsong and human speech. *Nature Communications* 10, 3636. doi:10.1038/s41467-019-11605-y

Spehar, B., Walker, N., and Taylor, R. (2016). Taxonomy of individual variations in aesthetic responses to fractal patterns. *Frontiers in Human Neuroscience* 10, 350. doi:10.3389/fnhum.2016.00350

Taylor, R. (2002). Order in Pollock's chaos - computer analysis is helping to explain the appeal of Jackson Pollock's paintings. *Scientific American* 287, 116–121

Wendt, H. and Abry, P. (2007). Multifractality tests using bootstrapped wavelet leaders. *IEEE Transactions on Signal Processing* 55, 4811–4820. doi:10.1109/TSP.2007.896269

Wendt, H., Roux, S. G., Jaffard, S., and Abry, P. (2009). Wavelet leaders and bootstrap for multifractal analysis of images. *Signal Processing* 89, 1100–1114. doi:10.1016/j.sigpro.2008.12.015

SUPPLEMENTARY TABLES AND FIGURES

Table S1: List of texts in Jena Corpus of Expository and Fictional Prose (JEFP Corpus). Canonical texts were selected from the Corpus of Canonical Western Literature. Non-canonical texts were downloaded from www.smashwords.com, www.goodreads.com, www.feedbooks.com, or Project Gutenberg. Non-fictional texts were sampled from Project Gutenberg.

	Title	Author(s)	Year of Publication	Category
1	Little Dorrit	Charles Dickens	1857	Canonical
2	Oliver Twist	Charles Dickens	1839	Canonical
3	The Life and Adventures of Nicholas Nickleby	Charles Dickens	1839	Canonical
4	The Mystery of Edwin Drood	Charles Dickens	1870	Canonical
5	The Pickwick Papers	Charles Dickens	1836	Canonical
6	Jane Eyre	Charlotte Bronte	1847	Canonical
7	Villette	Charlotte Bronte	1853	Canonical
8	Cranford	Elizabeth Gaskell	1853	Canonical
9	Mary Barton	Elizabeth Gaskell	1848	Canonical
10	North and South	Elizabeth Gaskell	1854	Canonical
11	Agnes Grey	Anne Bronte	1847	Canonical
12	Adam Bede	George Eliot	1859	Canonical
13	Daniel Deronda	George Eliot	1876	Canonical
14	Middlemarch	George Eliot	1872	Canonical
15	Silas Marner	George Eliot	1861	Canonical
16	The Mill on the Floss	George Eliot	1860	Canonical
17	Emma	Jane Austen	1815	Canonical
18	Mansfield Park	Jane Austen	1814	Canonical
19	Persuasion	Jane Austen	1818	Canonical
20	Pride and Prejudice	Jane Austen	1813	Canonical
21	The Picture of Dorian Gray	Oscar Wilde	1890	Canonical
22	The Tenant of Wildfell Hall	Anne Bronte	1848	Canonical
23	Sartor Resartus	Thomas Carlyle	1834	Canonical
24	Old Mortality	Walter Scott	1816	Canonical
25	Redgauntlet	Walter Scott	1824	Canonical
26	The Heart of Midlothian	Walter Scott	1818	Canonical
27	Waverley	Walter Scott	1814	Canonical
28	No Name	Wilkie Collins	1862	Canonical
29	The Moonstone	Wilkie Collins	1868	Canonical
30	The Woman in White	Wilkie Collins	1859	Canonical
31	The History of Henry Esmond	William Makepeace Thackeray	1852	Canonical
32	Vanity Fair	William Makepeace Thackeray	1847	Canonical
33	Dracula	Bram Stoker	1897	Canonical
34	The Well at the World's end	William Morris	1896	Canonical
35	The Narrative of Arthur Gordon Pym	Edgar Allan Poe	1838	Canonical
36	The Ambassadors	Henry James	1903	Canonical
37	The Awkward Age	Henry James	1899	Canonical
38	The Bostonians	Henry James	1886	Canonical
39	The Golden Bowl	Henry James	1904	Canonical
40	The Portrait of a Lady	Henry James	1881	Canonical
41	The Wings of Dove	Henry James	1902	Canonical

Continued on next page

Table S1 – *Continued from previous page*

	Title	Author(s)	Year of Publication	Category
42	Moby Dick	Herman Melville	1851	Canonical
43	The Deerslayers	James Fenimore Cooper	1841	Canonical
44	A Christmas Carol	Charles Dickens	1843	Canonical
45	Little Women	Louisa May Alcott	1868	Canonical
46	Puddnhead Wilson	Mark Twain	1893	Canonical
47	The Adventures of Finn	Mark Twain	1884	Canonical
48	The Mysterious Stranger	Mark Twain	1916	Canonical
49	The Marble Faun	Nathaniel Hawthorne	1859	Canonical
50	The Scarlet Letter	Nathaniel Hawthorne	1850	Canonical
51	The Education of Adams	Henry Adams	1907	Canonical
52	Walden	Henry David Thoreau	1854	Canonical
53	A Conneticut Yankee in King Arthurs	Mark Twain	1889	Canonical
54	Babbitt	Sinclair Lewis	1922	Canonical
55	A Tale of Two Cities	Charles Dickens	1859	Canonical
56	Sister Carrie	Theodore Dreiser	1900	Canonical
57	My Antonia	Willa Cather	1918	Canonical
58	The Old Wives Tale	Arnold Bennett	1908	Canonical
59	Portrait of the Artist as a Young Man	James Joyce	1916	Canonical
60	Ulysses	James Joyce	1922	Canonical
61	Lord Jim	Joseph Conrad	1900	Canonical
62	Nostromo	Joseph Conrad	1904	Canonical
63	The Secret Agent	Joseph Conrad	1907	Canonical
64	Under Western Eyes	Joseph Conrad	1911	Canonical
65	Victory: An Island Tale	Joseph Conrad	1915	Canonical
66	Bleak House	Charles Dickens	1853	Canonical
67	The Rainbow	Lawrence D.H	1915	Canonical
68	Women in Love	Lawrence D.H	1920	Canonical
69	Kim	Rudyard Kipling	1901	Canonical
70	Puck of Pooks Hill	Rudyard Kipling	1906	Canonical
71	Jude the Obscure	Thomas Hardy	1895	Canonical
72	Tess of the dUrbervilles	Thomas Hardy	1891	Canonical
73	The Mayor of Casterbridge	Thomas Hardy	1886	Canonical
74	The Return of the Native	Thomas Hardy	1878	Canonical
75	David Copperfield	Charles Dickens	1850	Canonical
76	Great Expectations	Charles Dickens	1860	Canonical
77	Hard Times	Charles Dickens	1854	Canonical
78	The Face in the Abyss	Abraham Merritt	1923	Non-Canonical
79	A Prisoner in Fairyland	Algernon Blackwood	1913	Non-Canonical
80	The Centaur	Algernon Blackwood	1911	Non-Canonical
81	Ruth Fielding at the War Front	Alice B. Emerson	1918	Non-Canonical
82	The International Spy	Allen Upward	1904	Non-Canonical
83	A Texas Matchmaker	Andy Adams	1904	Non-Canonical
84	The Filigree Ball	Anna Katharine Green	1903	Non-Canonical
85	Looking Further Backward	Arthur Dudley Vinton	1890	Non-Canonical
86	The Hill Of Dreams	Arthur Machen	1907	Non-Canonical
87	The Elusive Pimpernel	Baroness Emma Orczy	1908	Non-Canonical
88	The Gloved Hand	Burton E. Stevenson	1913	Non-Canonical
89	Jean of the Lazy A	B.M . Bower	1915	Non-Canonical
90	Wieland : or , The Transformation	Charles Brockden Brown	1798	Non-Canonical

Continued on next page

Table S1 – *Continued from previous page*

	Title	Author(s)	Year of Publication	Category
91	The Great Quest	Charles Hawes	1921	Non-Canonical
92	The Filibusters	Charles John Cutcliffe Wright Hyne	1900	Non-Canonical
93	Bar-20 Days	Clarence E. Mulford	1911	Non-Canonical
94	Wunpost	Dane Coolidge	1920	Non-Canonical
95	The Girl of the Golden West	David Belasco	1911	Non-Canonical
96	Love Insurance	Earl Derr Biggers	1914	Non-Canonical
97	The Wouldbegoods	Edith Nesbit	1899	Non-Canonical
98	Wet Magic	Edith Nesbit	1913	Non-Canonical
99	Philip Dru : Administrator	Edward Mandell House	1912	Non-Canonical
100	An Amiable Charlatan	Edward Phillips Oppenheim	1916	Non-Canonical
101	The Double Traitor	Edward Phillips Oppenheim	1915	Non-Canonical
102	The Zeppelin 's Passenger	Edward Phillips Oppenheim	1918	Non-Canonical
103	The People of the Ruins	Edward Shanks	1920	Non-Canonical
104	The Honor of the Name	Emile Gaboriau	1891	Non-Canonical
105	Kai Lung's Golden Hours	Ernest Bramah Smith	1922	Non-Canonical
106	The Riddle of the Sands	Erskine Childers	1903	Non-Canonical
107	The Missourian	Eugene Percy Lyle	1905	Non-Canonical
108	Privy Seal	Ford Madox Ford	1907	Non-Canonical
109	The Ivory Snuff Box	Frederic Arnold Kummer	1912	Non-Canonical
110	The Afterglow	George Allan England	1913	Non-Canonical
111	The Flying Legion	George Allan England	1920	Non-Canonical
112	West Wind Drift	George Barr McCutcheon	1920	Non-Canonical
113	Peter the Brazen	George F. Worts	1919	Non-Canonical
114	Olga Romanoff or , The Syren of the Skies	George Griffith	1894	Non-Canonical
115	The Princess and Curdie	George MacDonald	1883	Non-Canonical
116	The Adventures of Don Lavington	George Manville Fenn	1896	Non-Canonical
117	A Voyage to the Moon	George Tucker	1827	Non-Canonical
118	Claim Number One	George W. Ogden	1922	Non-Canonical
119	The Flockmaster of Poison Creek	George W. Ogden	1921	Non-Canonical
120	Trilby	George du Maurier	1894	Non-Canonical
121	Rose O'Paradise	Grace Miller White	1915	Non-Canonical
122	Condemned as a Nihilist	G. A. Henty	1893	Non-Canonical
123	Man on the Box	Harold MacGrath	1904	Non-Canonical
124	The Puppet Crown	Harold MacGrath	1901	Non-Canonical
125	The Blind Spot	Homer Eon Flint	1921	Non-Canonical
126	Men of Iron	Howard Pyle	1891	Non-Canonical
127	The Dark House	Ida Alexa Ross Wylie	1922	Non-Canonical
128	The Daughter of Brahma	Ida Alexa Ross Wylie	1912	Non-Canonical
129	Towards Morning	Ida Alexa Ross Wylie	1918	Non-Canonical
130	Jurgen : A Comedy of Justice	James Branch Cabell	1919	Non-Canonical
131	A Strange Manuscript Found in a Copper Cylinder	James De Mille	1888	Non-Canonical
132	Lost in the Fog	James De Mille	1870	Non-Canonical
133	Varney the Vampire	James Malcom Rymer	1847	Non-Canonical
134	The Danger Trail	James Oliver Curwood	1910	Non-Canonical
135	The Lost Stradivarius	John Meade Falkner	1895	Non-Canonical
136	The Nebuly Coat	John Meade Falkner	1903	Non-Canonical
137	The Weapons of Mystery	Joseph Hocking	1890	Non-Canonical
138	The Chestermarke Instinct	Joseph Smith Fletcher	1921	Non-Canonical

Continued on next page

Table S1 – *Continued from previous page*

	Title	Author(s)	Year of Publication	Category
139	Afloat On The Flood	Lawrence J. Leslie	1915	Non-Canonical
140	Diane of the Green Van	Leona Dalrymple	1914	Non-Canonical
141	Don Rodriguez : Chronicles of Shadow Valley	Lord Dunsany	1922	Non-Canonical
142	The Treasure Trail	Marah Ellis Ryan	1918	Non-Canonical
143	Mizora : A Prophecy	Mary E. Bradley	1889	Non-Canonical
144	Dangerous Days	Mary Roberts Rinehart	1919	Non-Canonical
145	The Blue Germ	Maurice Nicoll	1918	Non-Canonical
146	The Night Horseman	Max Brand	1920	Non-Canonical
147	The Sleuth of St. James 's Square	Melville Davisson Post	1920	Non-Canonical
148	Across the Zodiac	Percy Greg	1880	Non-Canonical
149	Bardelys the Magnificent	Rafael Sabatini	1905	Non-Canonical
150	Soldiers of Fortune	Richard Harding Davis	1897	Non-Canonical
151	The Beetle	Richard Marsh	1897	Non-Canonical
152	The Triumphs of Eugne Valmont	Robert Barr	1906	Non-Canonical
153	Dawn of All	Robert Hugh Benson	1911	Non-Canonical
154	Erling the Bold	Robert Michael Ballantyne	1869	Non-Canonical
155	The Dog Crusoe and His Master	Robert Michael Ballantyne	1894	Non-Canonical
156	Ailsa Paige	Robert William Chambers	1910	Non-Canonical
157	In Search of the Unknown	Robert William Chambers	1904	Non-Canonical
158	In the Quarter	Robert William Chambers	1894	Non-Canonical
159	Under the Ocean to the South Pole	Roy Rockwood	1907	Non-Canonical
160	Erewhon , or Over The Range	Samuel Butler	1910	Non-Canonical
161	The road to Frontenac	Samuel Merwin	1901	Non-Canonical
162	Brood of the Witch-Queen	Sax Rohmer	1918	Non-Canonical
163	The Revolt of Man	Walter Besant	1882	Non-Canonical
164	The Brass Bottle	Thomas Anstey Guthrie	1900	Non-Canonical
165	The Stray Lamb	Thorne Smith	1929	Non-Canonical
166	The Doomsman	Van Tassel Sutphen	1906	Non-Canonical
167	The Song of the Lark	Willa Cather	1915	Non-Canonical
168	The Old Tobacco Shop	William Bowen	1921	Non-Canonical
169	The Boats of the 'Glen-Carrig '	William Hope Hodgson	1907	Non-Canonical
170	Hushed Up!	William Le Queux	1911	Non-Canonical
171	The Border Legion	Zane Grey	1916	Non-Canonical
172	The Desert of Wheat	Zane Grey	1919	Non-Canonical
173	Scottish Cathedrals and Abbeys	Dugald Butler	1901	Non-Fictional
174	A Text-Book of the History of Architecture: Seventh Edition, revised	A. D. F. Hamlin	1896	Non-Fictional
175	Some Account of Gothic Architecture in Spain	George Edmund Street	1865	Non-Fictional
176	Japanese Homes and Their Surroundings	Edward Sylvester Morse	1885	Non-Fictional
177	The Architecture of Provence and the Riviera	David MacGibbon	1888	Non-Fictional
178	Historic Ornament, Vol. 2: Treatise on decorative art and architectural ornament	James Ward	1897	Non-Fictional
179	Military Architecture in England During the Middle Ages	A. Hamilton Thompson	1912	Non-Fictional
180	How to Study Architecture	Charles H. Caffin	1917	Non-Fictional
181	Cakes & Ale: A Dissertation on Banquets Interspersed with Various Recipes, More or Less Original, and anecdotes, mainly veracious	Edward Spencer	1897	Non-Fictional
182	Food and Flavor: A Gastronomic Guide to Health and Good Living	Henry T. Finck	1913	Non-Fictional

Continued on next page

Table S1 – *Continued from previous page*

	Title	Author(s)	Year of Publication	Category
183	A Concise Dictionary of Middle English from A.D. 1150 to 1580	A. L. Mayhew, Walter William Skeat	1888	Non-Fictional
184	A Dictionary of Slang, Cant, and Vulgar Words: Used at the Present Day in the Streets of London	John Camden Hotten	1860	Non-Fictional
185	The Devil's Dictionary	Ambrose Bierce	1906	Non-Fictional
186	The Encyclopedia Britannica Vol. 1	University of Cambridge	1910	Non-Fictional
187	The Encyclopedia Britannica Vol. 2	University of Cambridge	1910	Non-Fictional
188	Glossary of Chess terms	Gregory Zorzos	2017	Non-Fictional
189	Through the Brazilian Wilderness	Roosevelt	1914	Non-Fictional
190	Gold, Sport, and Coffee Planting in Mysore	Robert H. Elliot	1898	Non-Fictional
191	The Economic Aspect of Geology	C. K. Leith	1921	Non-Fictional
192	The Shores of the Adriatic: The Austrian Side, The Kustenlande, Istria, and Dalmatia	F. Hamilton Jackson	1906	Non-Fictional
193	Island Life; Or, The Phenomena and Causes of Insular Faunas and Floras	Alfred Russel Wallace	1880	Non-Fictional
194	Sea and Sardinia	D. H. Lawrence	1921	Non-Fictional
195	Sketches from the Subject and Neighbour Lands of Venice	Edward A. Freeman	1881	Non-Fictional
196	The Elements of Geology	William Harmon Norton	1905	Non-Fictional
197	The Principles of Stratigraphical Geology	J. E. Marr	1898	Non-Fictional
198	Fragments of Earth Lore: Sketches & Addresses Geological and Geographical	James Geikie	1893	Non-Fictional
199	Earth Features and Their Meaning: An Introduction to Geology for the Student and the General Reader	William Herbert Hobbs	1912	Non-Fictional
200	The Common Law	Oliver Wendell Holmes	1881	Non-Fictional
201	Babylonian and Assyrian Laws, Contracts and Letters	C. H. W. Johns	1904	Non-Fictional
202	Putnam's Handy Law Book for the Layman	Albert Sidney Bolles	1921	Non-Fictional
203	Marriage and Divorce Laws of the World	Hyacinthe Ringrose	1911	Non-Fictional
204	The Law and the Poor	Edward Abbott Parry	1914	Non-Fictional
205	International Law. A Treatise. Vol. 1: Peace. Second Edition	L. Oppenheim	1905	Non-Fictional
206	International Law. A Treatise. Vol. 2: War and Neutrality. Second Edition	L. Oppenheim	1905	Non-Fictional
207	International Law	George Grafton Wilson, George Fox Tucker	1901	Non-Fictional
208	The Criminal Prosecution and Capital Punishment of Animals	E. P. Evans	1906	Non-Fictional
209	The English Constitution	Walter Bagehot	1867	Non-Fictional
210	The Law of the Sea: A Manual of the Principles of Admiralty Law for Students, Mariners, and Ship Operators	George L. Canfield, George W. Dalzell, J. Y. Brinton	1921	Non-Fictional
211	Woman and the Republic: A Survey of the Woman-Suffrage Movement in the United States and a Discussion of the Claims and Arguments of Its Foremost Advocates	Helen Kendrick Johnson	1897	Non-Fictional
212	The American Judiciary	Simeon E. Baldwin	1905	Non-Fictional
213	The Story of Evolution	Joseph McCabe	1912	Non-Fictional
214	A Practical Physiology: A Text-Book for Higher Schools	Albert F. Blaisdell	1897	Non-Fictional
215	Our Vanishing Wild Life: Its Extermination and Preservation	William T. Hornaday	1913	Non-Fictional
216	Amusements in Mathematics	Henry Ernest Dudeney	1917	Non-Fictional
217	On the Genesis of Species	St. George Jackson Mivart	1871	Non-Fictional
218	An Elementary Study of Chemistry	William McPherson, William Edwards Henderson	1906	Non-Fictional
219	Great Astronomers	Robert S. Ball	1895	Non-Fictional
220	Evolution, Old & New	Samuel Butler	1879	Non-Fictional

Continued on next page

Table S1 – *Continued from previous page*

	Title	Author(s)	Year of Publication	Category
221	Darwin, and After Darwin, Vol. 1: An Exposition of the Darwinian Theory and a Discussion of Post-Darwinian Questions	George John Romanes	1982	Non-Fictional
222	Creative Evolution	Henri Bergson	1907	Non-Fictional
223	Myths and Marvels of Astronomy	Richard A. Proctor	1877	Non-Fictional
224	A Popular History of Astronomy During the Nineteenth Century: Fourth Edition	Agnes M. Clerke	1887	Non-Fictional
225	Pioneers of Science	Oliver Lodge	1893	Non-Fictional
226	A Text-Book of Astronomy	George C. Comstock	1901	Non-Fictional
227	Astronomical Myths: Based on Flammarions's "History of the Heavens"	Camille Flammarion, J. F. Blake	1877	Non-Fictional
228	Darwin, and After Darwin, Vol. 2: Post-Darwinian Questions, Heredity and Utility	George John Romanes	1892	Non-Fictional
229	Astronomy: The Science of the Heavenly Bodies	David P. Todd	1922	Non-Fictional
230	The Foundations of Science: Science and Hypothesis, The Value of Science, Science and Method	Henri Poincaré	1913	Non-Fictional
231	A Civic Biology, Presented in Problems	George W. Hunter	1914	Non-Fictional
232	Physics	Willis E. Tower, Charles M. Turton, Charles H. Smith, Thomas D. Cope	1920	Non-Fictional
233	A Century of Science, and Other Essays	John Fiske	1899	Non-Fictional
234	Side-Lights on Astronomy and Kindred Fields of Popular Science	Simon Newcomb	1906	Non-Fictional
235	Elementary Zoology, Second Edition	Vernon L. Kellogg	1901	Non-Fictional
236	Experiments on Animals	Stephen Paget	1888	Non-Fictional
237	The Sea-beach at Ebb-tide: A Guide to the Study of the Seaweeds and the Lower Animal Life Found Between Tide-marks	Augusta Foote Arnold	1901	Non-Fictional
238	The Making of Species	Douglas Dewar, Frank Finn	1909	Non-Fictional
239	The Science and Philosophy of the Organism	Hans Driesch	1908	Non-Fictional
240	Problems of Genetics	William Bateson	1913	Non-Fictional
241	The Organism as a Whole, from a Physicochemical Viewpoint	Jacques Loeb	1916	Non-Fictional
242	A Guide to the Study of Fishes, Vol. 1	David Starr Jordan	1905	Non-Fictional
243	Evolution: Its nature, its evidence, and its relation to religious thought	Joseph LeConte	1888	Non-Fictional
244	The Races of Man: An Outline of Anthropology and Ethnography	Joseph Deniker	1900	Non-Fictional
245	Physiology: The Science of the Body	Ernest G. Martin	1922	Non-Fictional
246	Observations of a Naturalist in the Pacific Between 1896 and 1899, Vol. 1	H. B. Guppy	1903	Non-Fictional
247	Animal Life and Intelligence	C. Lloyd Morgan	1890	Non-Fictional
248	A Guide to the Study of Fishes, Vol. 2	David Starr Jordan	1905	Non-Fictional
249	Stargazing: Past and Present	Norman Lockyer	1878	Non-Fictional
250	Observations of a Naturalist in the Pacific Between 1896 and 1899, Vol. 2	H. B. Guppy	1903	Non-Fictional
251	Regeneration	Thomas Hunt Morgan	1901	Non-Fictional
252	Telescopic Work for Starlight Evenings	William F. Denning	1891	Non-Fictional
253	The Logic of Chance, 3rd edition	John Venn	1888	Non-Fictional
254	Biology and Its Makers: With Portraits and Other Illustrations	William A. Locy	1908	Non-Fictional
255	The Crayfish: An Introduction to the Study of Zoology	Thomas Henry Huxley	1880	Non-Fictional

Continued on next page

Table S1 – *Continued from previous page*

	Title	Author(s)	Year of Publication	Category
256	History of Botany (1530-1860)	Julius Sachs	1875	Non-Fictional
257	The Universal Kinship	J. Howard Moore	1906	Non-Fictional
258	The philosophy of biology	James Johnstone	1914	Non-Fictional
259	Hygienic Physiology: with Special Reference to the Use of Alcoholic Drinks and Narcotics	Joel Dorman Steele	1884	Non-Fictional
260	Species and Varieties, Their Origin by Mutation	Hugo de Vries	1905	Non-Fictional
261	The Naturalist in La Plata	W. H. Hudson	1892	Non-Fictional
262	Studies in the Psychology of Sex, Vol. 1	Havelock Ellis	1900	Non-Fictional
263	Studies in the Psychology of Sex, Vol. 2	Havelock Ellis	1900	Non-Fictional
264	The Mind of the Child, Part II: The Development of the Intellect	William T. Preyer	1888	Non-Fictional
265	The Measurement of Intelligence	Lewis M. Terman	1916	Non-Fictional
266	Human Traits and their Social Significance	Irwin Edman	1919	Non-Fictional
267	Chapters in the History of the Insane in the British Isles	Daniel Hack Tuke	1882	Non-Fictional
268	Human Personality and Its Survival of Bodily Death	F. W. H. Myers	1903	Non-Fictional
269	Mysterious Psychic Forces: An Account of the Author's Investigations in Psychical Research, Together with Those of Other European Savants	Camille Flammarion	1907	Non-Fictional
270	The Group Mind: A Sketch of the Principles of Collective Psychology	William McDougall	1920	Non-Fictional
271	On the State of Lunacy and the Legal Provision for the Insane: With Observations on the Construction and Organization of Asylums	J. T. Arlidge	1859	Non-Fictional
272	The Criminal	Havelock Ellis	1890	Non-Fictional
273	Fact and Fable in Psychology	Joseph Jastrow	1900	Non-Fictional
274	Mental Evolution in Man: Origin of Human Faculty	George John Romanes	1888	Non-Fictional
275	A Beginner's Psychology	Edward Bradford Titchener	1915	Non-Fictional
276	Mental diseases: A Public Health Problem	James Vance May	1922	Non-Fictional
277	The Law of Psychic Phenomena	Thomson Jay Hudson	1893	Non-Fictional
278	Psychology: Briefer Course	William James	1892	Non-Fictional
279	The Principles of Psychology, Vol. 1	William James	1890	Non-Fictional
280	The Principles of Psychology, Vol. 2	William James	1890	Non-Fictional
281	Sex & Character	Otto Weininger	1906	Non-Fictional
282	Youth: Its Education, Regimen, and Hygiene	G. Stanley Hall	1906	Non-Fictional
283	Ten Thousand Dreams Interpreted; Or, What's in a Dream: A Scientific and Practical Exposition	Gustavus Hindman Miller	1906	Non-Fictional
284	Browning as a Philosophical and Religious Teacher	Henry Jones	1891	Non-Fictional
285	The Life of Reason: The Phases of Human Progress	George Santayana	1905	Non-Fictional
286	An Introduction to Philosophy	George Stuart Fullerton	1906	Non-Fictional
287	The Approach to Philosophy	Ralph Barton Perry	1905	Non-Fictional
288	The Will to Believe, and Other Essays in Popular Philosophy	William James	1896	Non-Fictional
289	Christianity and Greek Philosophy	B. F. Cocker	1870	Non-Fictional
290	A History of Mediaeval Jewish Philosophy	Isaac Husik	1916	Non-Fictional
291	The Mediaeval Mind (Vol. 1 of 2): A History of the Development of Thought and Emotion in the Middle Ages	Henry Osborn Taylor	1911	Non-Fictional
292	The Mediaeval Mind (Vol. 2 of 2): A History of the Development of Thought and Emotion in the Middle Ages	Henry Osborn Taylor	1911	Non-Fictional
293	The Philosophy of Friedrich Nietzsche	H. L. Mencken	1908	Non-Fictional
294	Philosophical Studies	G. E. Moore	1883	Non-Fictional
295	What Nietzsche Taught	Willard Huntington Wright	1915	Non-Fictional
296	The Greek Philosophers, Vol. 1	Alfred William Benn	1882	Non-Fictional

Continued on next page

Table S1 – *Continued from previous page*

	Title	Author(s)	Year of Publication	Category
297	The Greek Philosophers, Vol. 2	Alfred William Benn	1882	Non-Fictional
298	An Ethical Philosophy of Life Presented in Its Main Outlines	Felix Adler	1918	Non-Fictional
299	A Beginner's History of Philosophy, Vol. 1	Herbert Ernest Cushman	1910	Non-Fictional
300	Towards the Great Peace	Ralph Adams Cram	1922	Non-Fictional
301	Society: Its Origin and Development	Henry K. Rowe	1916	Non-Fictional
302	Criminal Man, According to the Classification of Cesare Lombroso	Gina Lombroso	1880	Non-Fictional
303	The Challenge of the Country: A Study of Country Life Opportunity	George Walter Fiske	1912	Non-Fictional
304	Criminal Sociology	Enrico Ferri	1895	Non-Fictional
305	Community Civics and Rural Life	Arthur William Dunn	1920	Non-Fictional
306	Sociology and Modern Social Problems	Charles A. Ellwood	1910	Non-Fictional
307	The Theory of the Leisure Class	Thorstein Veblen	1899	Non-Fictional

Table S2: $Means(\pm SD)$ of the different text properties analyzed in the present study. Abbreviation: MTLD, Measure for Textual Lexical Diversity. Asterisks indicate that results are different from fictional and canonical texts, respectively, at *, $p < 0.05$; **, $p < 0.01$; and ***, $p < 0.001$ (paired t-tests).

	Fictional	Non-Fictional	Canonical	Non-Canonical
Noun	3.80 (1.21)	5.76 (1.21)***	4.11 (1.24)	3.55 (1.11)
Verb	3.30 (0.90)	3.22 (0.96)	3.54 (0.83)	3.10 (0.90)
Adjective	1.28 (0.48)	2.06 (0.64)***	1.45 (0.45)	1.13 (0.45)*
Pronoun	2.04 (0.57)	1.01 (0.49)***	2.25 (0.57)	1.86 (0.50)*
Sentence-Length	20.91 (6.15)	25.13 (6.18)***	22.82 (5.83)	19.37 (5.96)*
MTLD	48.34 (7.44)	45.43 (8.56)**	47.81 (6.08)	48.76 (8.36)
Topic Distribution	0.70 (0.02)	0.68 (0.03)***	0.70 (0.02)	0.71 (0.03)

Table S3: Accuracy of classification (in %) using the mean value of the text properties for the non-fictional/fictional distinction (Task 1) and the canonical/non-canonical distinction (Task 2). $Means \pm SD$ are listed (N = 10). All values are significantly different ($p \leq 0.05$) from random accuracy (50%), except where indicated by a †.

	Task 1	Task 2
Noun	83.0 ± 2.2	56.5 ± 3.8
Verb	53.1 ± 2.0	63.8 ± 3.8
Adjective	76.3 ± 2.3	70.5 ± 3.2
Pronoun	81.3 ± 2.8	65.5 ± 3.0
Sentence-Length	66.5 ± 2.8	62.6 ± 3.5
MTLD	51.2 ± 1.7†	55.6 ± 4.0
Topic Distribution	76.3 ± 2.0	51.7 ± 2.4†
Low-Level	96.5 ± 1.0	66.9 ± 3.6
High-Level	79.5 ± 2.0	57.2 ± 3.3
Low- & High-Level	96.7 ± 1.0	73.5 ± 1.7

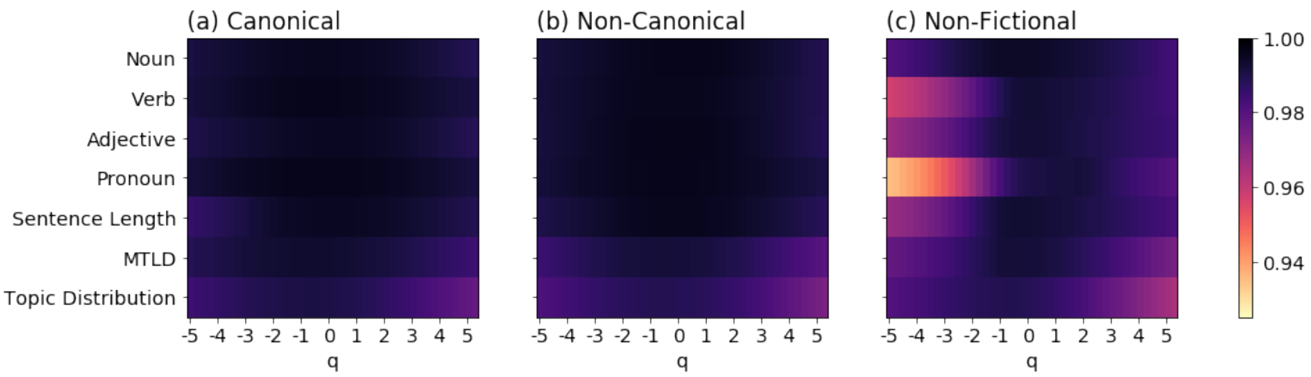

Figure S1: Mean R^2 of the linear fits to the fluctuation function of the MFDFA method for different values of q and for different text properties in canonical (a), non-canonical (b) and non-fictional texts (c) in the corpus. The color coding is shown on the right hand side.

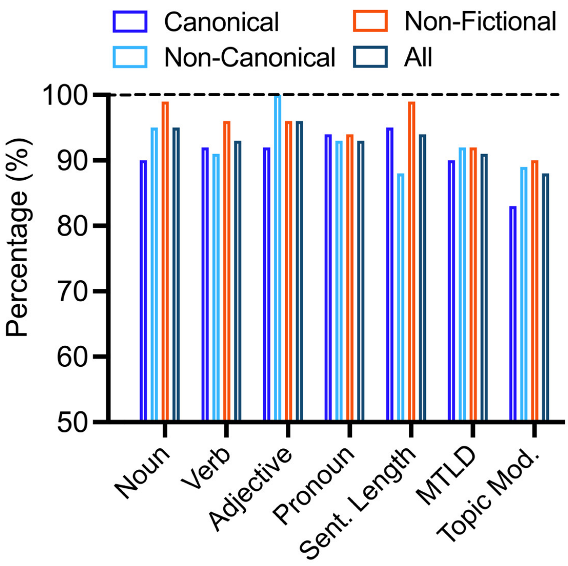

Figure S2: The percentage of the texts for which the degree of multifractality is significantly larger than the degree of multifractality of their surrogates ($p < 0.05$). The different text properties are indicated at the bottom. The color of the bars represent the categories of text separately and all together, as shown on top of the figure.
